# Action planning and control under uncertainty emerge through a desirability-driven competition between parallel encoding motor plans

**DOI:** 10.1371/journal.pcbi.1009429

**Published:** 2021-10-01

**Authors:** Vince Enachescu, Paul Schrater, Stefan Schaal, Vassilios Christopoulos

**Affiliations:** 1 Department of Neuroscience, University of Southern California, Los Angeles, California, United States of America; 2 Department of Computer Science, University of Southern California, Los Angeles, California, United States of America; 3 Department of Computer Science and Engineering, University of Minnesota, Minneapolis, Minnesota, United States of America; 4 Department of Psychology, University of Minnesota, Minneapolis, Minnesota, United States of America; 5 Department of Bioengineering, University of California Riverside, Riverside, California, United States of America; Durham University, UNITED KINGDOM

## Abstract

Living in an uncertain world, nearly all of our decisions are made with some degree of uncertainty about the consequences of actions selected. Although a significant progress has been made in understanding how the sensorimotor system incorporates uncertainty into the decision-making process, the preponderance of studies focus on tasks in which selection and action are two separate processes. First people select among alternative options and then initiate an action to implement the choice. However, we often make decisions during ongoing actions in which the value and availability of the alternatives can change with time and previous actions. The current study aims to decipher how the brain deals with uncertainty in decisions that evolve while acting. To address this question, we trained individuals to perform rapid reaching movements towards two potential targets, where the true target location was revealed only after the movement initiation. We found that reaction time and initial approach direction are correlated, where initial movements towards intermediate locations have longer reaction times than movements that aim directly to the target locations. Interestingly, the association between reaction time and approach direction was independent of the target probability. By modeling the task within a recently proposed neurodynamical framework, we showed that action planning and control under uncertainty emerge through a desirability-driven competition between motor plans that are encoded in parallel.

## Author summary

Uncertainty is ubiquitous in our sensorimotor interaction with the external world, and motor decisions regularly have to be made in the face of it. Even after an action is selected, there is residual uncertainty, reflecting the subjective belief about whether the current choice is *better* compared to the alternative options. Over the past years, several experimental studies have reported that in situations affording more than one possible target of action, individuals delay their decision by moving towards an intermediate location between the potential targets, a strategy consistent with increasing chances of collecting more information before making a decision. This spatial averaging behavior suggests that the brain incorporates uncertainty into the planning and execution of actions. However, there is no strong consensus on how uncertainty is represented and resolved in the sensorimotor system, and affects motor decisions. By training people to perform rapid reaching movements in the presence of two potential targets, we provide evidence that goal location uncertainty is resolved within the action-space and through a desirability-driven competition between motor plans that are encoded in parallel.

## 1 Introduction

On January 15, 2009, the US Airways flight 1549, a domestic flight from La Guardia airport in New York City to Seattle, experienced a complete loss of thrust in both engines after encountering a flock of Canada geese. As the aircraft lost altitude, the air traffic control asked the pilot if he could either return to La Guardia or to land at the nearby Teterboro airport. Having less than 5 minutes after the bird strike to land the plane, the pilot had to evaluate the alternative options before making a final decision. This incident describes a ubiquitous situation, in which decisions made while acting and without having complete certainty about the outcomes of the alternative options. How uncertainty about the outcome of actions is represented into the sensorimotor system and influences motor behavior has, so far, remained elusive. In laboratory-based tasks when participants are faced with similar uncertainty about which option to select, they often choose a third (and occasionally lower rewarded) alternative option [1, 2]. In the plane example, the pilot followed a similar strategy by rejecting both options, because he was not certain that he could make any runway. Instead, he decided to safely glide the plane to ditch in the Hudson river, an action that saved the passengers’ lives. However, in situations in which a third option is not available, individuals often delay their choice by moving towards an intermediate location between the alternative options, waiting to accumulate more information before making a decision [3–11]. This spatial averaging behavior is thought to provide fundamental insight on how the sensorimotor system integrates uncertainty into planning and execution of actions to achieve the desired outcome. However, despite many years of research, there is no strong consensus about the mechanism that generates the spatial averaging behavior.

A critical issue impeding consensus is the locus of uncertainty. One family of theories suggests that the brain deals with goal location uncertainty by generating a single intermediate movement that is corrected online as more information becomes available [11–13]. Here, the locus of uncertainty is over the target space. If there are two potential targets for a reach, generating an intermediate movement allows individuals to come closer to both options, affording more time to determine the option with the best outcome before making a commitment to either one. Recent cognitive theories argue against this hypothesis proposing that in situations affording more than one alternative options, individuals plan in parallel multiple actions that compete for selection before choosing one to execute [6–8, 10]. This theory is known as “motor encoding strategy” and suggests that intermediate movements reflect a combination of distinct (i.e., single-target) motor actions that have been *partially* planned—only some aspects of the motor action, such as direction, are planned prior to movement initiation—for each competing option [14]. The key concept in this theory is that decision and action are not separated and serial processes, rather action selection forms an integral part of the decision making process [15]. Importantly, the locus of uncertainty is over the set of motor plans.

A series of studies attempted to decipher whether the spatial averaging behavior reflects a single intermediate movement plan or a combination of single-target movement plans. In one of them, people had to initiate reaching movements towards two potential targets, while the true target location was revealed only after the movement onset [11]. The study reported that spatial averaging occurs only for slow movements, when time allows for corrective movements to be made. In contrast, fast movements were almost exclusively aimed directly to one of the two targets. These results were interpreted as evidence that the sensorimotor system plans and executes only a single, flexible motor plan, to optimize for uncertain goals [11]. However, another recent study used a similar “reach-before-you-know” paradigm and showed that if an obstacle blocked the movement towards one of the two targets, the intermediate movement was biased away from the obstacle. Because the location of the obstacle does not affect movements towards an averaged visual-spatial target location, these results appear to favor motor encoding strategy over planning a single intermediate movement [16].

To understand the mechanism of goal location uncertainty in motor decisions, we need to decipher what spatial averaging behavior reflects. Does the brain plan and execute a single motor plan prior to implementing a corrective action towards the cued target, or does it plan multiple single-target motor plans and execute a weighted average of them? To do so, we trained participants to perform rapid reaching movements to two potential targets presented simultaneously in both hemifields. Critically, the actual goal location was not disclosed before movement onset. Only after the reaching movement exceeded a threshold, the actual goal location was revealed. This ensured that the participants were not certain about the current best action before planning their movements, even after departing from the origin. Dual-target trials were interleaved with single-target trials in which one target was presented either in the left or the right hemifield. By varying the target probability, we tested how goal location uncertainty influences motor behavior. We found that when both targets had about the same probability, individuals delayed initiating a movement and aimed towards an intermediate location, waiting to collect more information before selecting one of the targets—the spatial averaging strategy reported in previous studies [6, 9, 12]. Alternatively, when one of the targets had higher probability, reaches had faster responses and launched closer to the likely target. These findings suggest that target probability influences both planning and execution of actions in motor decisions with multiple competing options. Surprisingly, the relationship between approach direction and reaction time was not mediated by the target probability. Instead, when people waited longer to initiate an action, reaches were frequently slower and launched towards an intermediate location between the potential goals, regardless of the target probability.

To better understand the association between reaction time and approach direction, we modeled the reaching task within a recently proposed computational theory [17, 18]. This theory is an extension of evidence accumulation models [19] and builds on the affordance competition hypothesis, in which multiple actions are formed concurrently and compete over time until one has sufficient evidence to win the competition [14, 20]. We replace evidence with desirability—a continuously evolving quantity that integrates all sources of information about the relative value of an action with respect to alternatives. It includes information not only about the uncertainty of goal location, but also about the effort required to perform an action at any given time and state. The relative desirability of an action varies with time and state and can be viewed as an internal estimate of our belief about the “attractiveness” of the action with respect to alternatives. Reaching movements are generated as a mixture of actions weighted by their relative desirability values. The difference of the desirability values at a given time and state characterizes the momentary degree of competition between the two alternative motor plans. Ambiguous desirabilities indicate that the net evidence supporting one action over the others is weak, and therefore the competition as to which action to select is high. On the contrary, when one action outperforms the alternatives, the net evidence is strong and therefore the action competition is low. In analogy with the evidence accumulation models [21–23], the action competition characterizes the momentary degree of choice uncertainty, such as the stronger the competition, the higher the uncertainty as to which of the two potential reach targets to act upon. Therefore, the “winning” action determines the selected target and the reaction time, whereas the “losing” actions contribute to the computation of uncertainty—i.e., the closer the desirability of the non-selected actions to the desirability of the selected one, the higher the uncertainty about the current best action. This is similar to the “balance-of-evidence” idea used in evidence accumulation models to determine choice uncertainty in perceptual judgment tasks [24]. Because desirability is time- and state- dependent, and action competition is often not resolved after movement onset, selected actions can be changed or corrected in-flight (i.e., change of mind) in the presence of new incoming information. The model predicts that both movement direction and reaction time are influenced by the relative desirabilities of the alternative actions. When the desirability values of the alternative actions are about the same, decisions are delayed by both moving towards an intermediate location and by having longer reaction time. In contrast, when one action has higher desirability and outperforms the alternatives, reaches are initiated faster and move directly to a target. Importantly, the model predicts that the association between approach direction and reaction time is not mediated by the target probability. Instead, action competition can increase the uncertainty about the best current action leading to slower responses regardless of target probability. Overall, model predictions are consistent with human findings suggesting that action planning and control under uncertainty emerge through a desirability-driven competition between motor plans that are encoded in parallel.

## 2 Results

### 2.1 Reaching strategies for dealing with goal uncertainty

**Fig 1:**
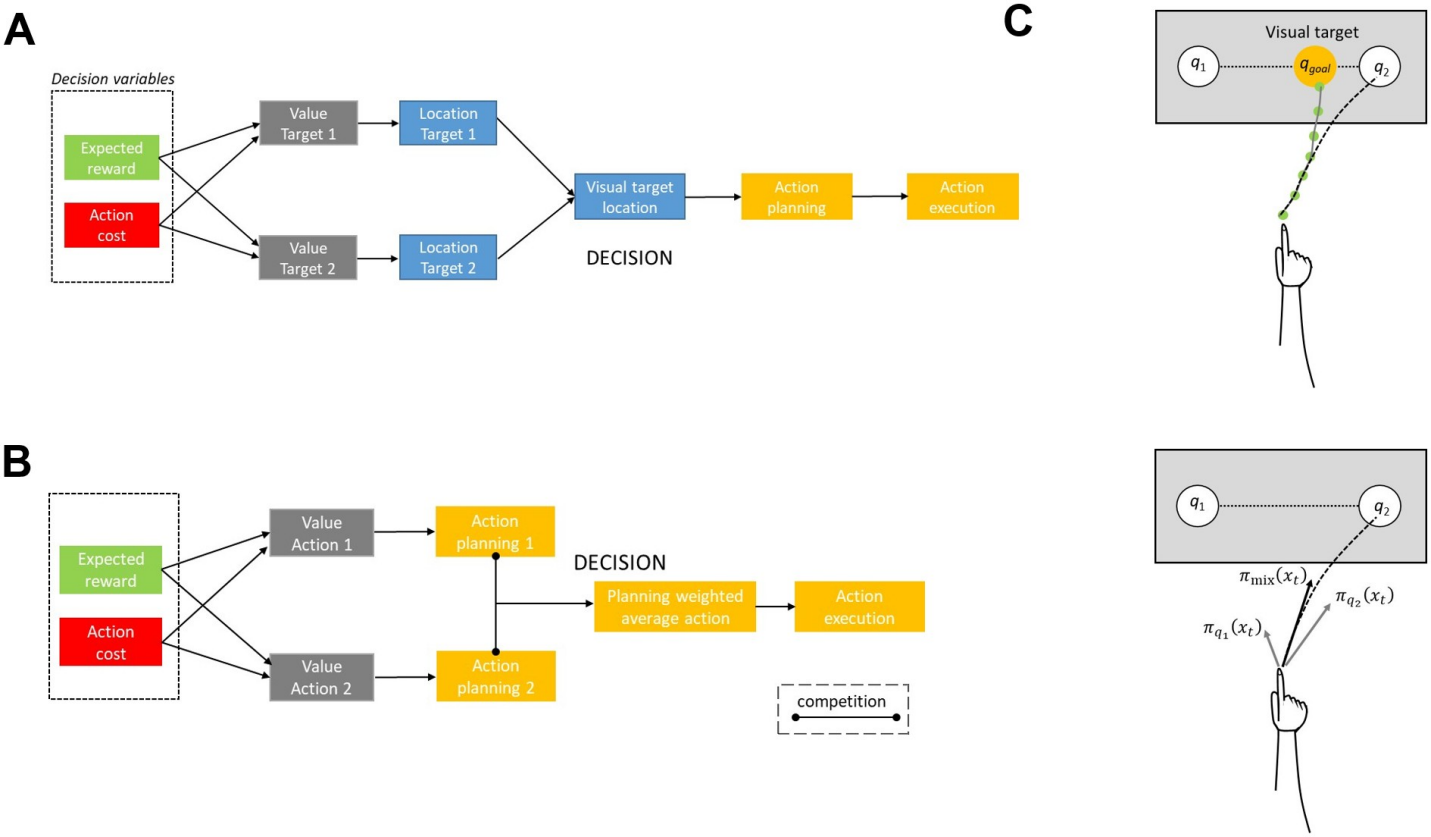
A graphical representation of two alternative hypotheses account spatial averaging behavior. (**A**): The visual encoding strategy suggests that the brain evaluates the potential targets based on the decision variables (e.g., expected reward and effort cost) and generates an averaged visual-spatial target location *q*_*goal*_(*x*_*t*_) (right panel). The “visual” target is computed as the weighted average of the two potential target locations (*q*_1_ and *q*_2_). The reach trajectory (gray continuous trace on the right panel) that aims towards the visual target location can be simulated using an optimal waypoint (green points) planning algorithm. Once the actual goal is revealed, the movement is corrected in-flight to the actual target location. Therefore, the reach trajectory consists of an initial phase towards the visual target location (gray trace) and a final phase towards the correct target location (black discontinuous trace). (**B**): The alternative hypothesis suggests that the brain plans simultaneously single-target actions (i.e., control policies) *π*_1_ and *π*_2_ that compete for selection and uses the decision variables to bias the action competition. The weighted average of the individuals planned single-target actions produces the motor action *π*_*mix*_ that generates the reaching movement (black discontinuous trace right panel).

A plethora of studies have showed that when encountering with situations in which there are multiple potential targets for action, individuals aim towards an intermediate location between the alternative options to compensate for goal uncertainty [6, 9, 12, 25]. Despite many years of research, there is no strong consensus on what this spatial averaging behavior reflects. One theory suggests that an intermediate trajectory reflects a “visual encoding” strategy that the brain plans to compensate for the goal location uncertainty [14, 25]. According to this theory, an averaged visual-spatial target location is planned and executed. A graphical representation of the visual encoding process is shown in Fig 1A (left panel). Decision variables, such as expected reward and action cost are integrated into the target *value* *V*. Then, a visual target is generated by averaging the locations of the two potential targets weighted by the corresponding target values—i.e., visual target is located closer to the target with the highest value. After a decision is made, an action towards the visual target location is planned and executed. The location of the visual target is continuously updated based on incoming information, e.g., changes of the target probability. Once one of the targets is cued, the trajectory is corrected in-flight to the actual goal location. The key characteristic of this theory is that decision takes place within the target space before an action is planned—the brain first specifies the visual target location and then plans an action to implement the choice. To illustrate the mathematical formulation of this theory, let’s consider two potential targets that are located at *q*_1_ and *q*_2_, and each of them is assigned with probability *P*_1_(*x*_*t*_) and *P*_2_(*x*_*t*_), and action cost *cost*_1_(*x*_*t*_) and *cost*_2_(*x*_*t*_)—i.e., effort to reach towards the target locations—at a given time and state *x*_*t*_, Fig 1A (right panel). If *V*_1_(*x*_*t*_) and *V*_2_(*x*_*t*_) are the normalized values of target 1 and target 2, respectively, such as *V*_1_(*x*_*t*_) + *V*_2_(*x*_*t*_) = 1, the visual average target location is computed as:

$$\begin{array}{c} q_{goal}\left(x_{t}\right)=q_{1}V_{1}\left(x_{t}\right)+q_{2}V_{2}\left(x_{t}\right) \end{array}$$ (1)

After selecting the goal location *q*_*goal*_, individuals need to specify the action plan (i.e., trajectory) to reach to that location. We can simulate this process using feedback control theory to generate a reach trajectory through optimal waypoints [26]. We define the discrete-time state-space representation of the reach trajectory as:

$$\begin{array}{ccc} x_{t+1} & = & A_{d}x_{t}+B_{d}u_{t} \end{array}$$ (2)

$$\begin{array}{ccc} y_{t} & = & C_{d}x_{t} \end{array}$$ (3)

where *x*_*t*_ is the state of the reaching movement, *A*_*d*_, *B*_*d*_ are the system dynamic matrices that describe how the current state *x*_*t*_ and the control inputs *u*_*t*_, respectively, affect the future state *x*_*t*+1_, and *C*_*d*_ is the observation matrix. Let’s assume $y_{k=1:M}^{w}$ be a set of M waypoints from the origin to the visual target location *q*_*goal*_. We can construct a discrete optimal control problem, such that the output *y*_*k*_ passes through the waypoints at specific time instances $y_{m_{i}}=y_{m_{i}}^{w}$. The waypoint planning and trajectory generation was used to emphasize that in visual averaging strategy a single intermediate movement is fully planned prior to movement initiation. Let the initial state be given by *x*(*t* = 0) = *x*_*init*_. The boundary condition at the end point is determined by the last waypoint, $y_{m_{k}}^{w}$ (i.e., the final destination, which is the target location *q*_*goal*_), such as $Cx_{m_{k}}=y_{m_{k}}^{w}$. The optimal control problem can be formulated as:

$$\begin{array}{c} J_{q_{goal}}\left(x,\pi ,y^{w}\right)=\frac{1}{2}\sum_{i=0}^{m_{k}}(x_{i}^{T}Qx_{i}+\pi {\left(x_{i}\right)}^{T}R\pi \left(x_{i}\right))+\frac{1}{2}\sum_{j=1}^{k}{\left(C_{d}x_{m_{j}}-y_{m_{j}}^{w}\right)}^{T}K(C_{d}x_{m_{j}}-y_{m_{j}}^{w}) \end{array}$$ (4)

where *Q*, *R* and *K* are square symmetric matrices. The minimization of Eq (4) provides the optimal policy *π*(*x*_*t*_), which is a set of actions $U^{q_{goal}}={\left[u_{1},u_{2},...,u_{m_{k}}\right]}^{T}$ that generate the trajectory through the *k* waypoints to reach to the visual target location *q*_*goal*_ (gray trace Fig 1A right panel). Since the actual goal location will be revealed after departing from the origin, the optimal policy needs to be recalculated after *n* steps, a strategy known as *receding horizon control* [27, 28]. Once the goal location is cued, the movement will be corrected in-flight to the actual goal. Therefore, a reach trajectory consists of an initial phase that aims towards the visual target location, and a final phase that aims to the correct target location (black trace in Fig 1A right panel). Along the same lines, another theory suggests that intermediate movement reflects a single, deliberate movement plan that is optimal given a partially formed belief about the true goal location [13]. When the strategic benefits of intermediate movement are mitigated by either reducing the time available to correct an action [13] or by increasing the spatial separation between the potential targets [11, 29], the spatial averaging behavior is largely abated [30].

An alternative theory argues against the single motor plan strategy, suggesting that the brain plans in parallel partially prepared actions that compete for selection Fig 1B (left panel) [10, 20, 31]. According to this theory, decision and action are coupled into a parallel process—multiple actions are *partially* prepared in parallel and compete for selection. Expected reward and action cost are integrated into the action value to bias the competition. A reaching movement is generated as a mixture of the single-target actions weighted by value of the actions. The key characteristic of this theory is that decision takes place in the action-space through action competition—i.e., the action that wins the competition determines the selected target and the reaction time. In a recent work, we modeled the motor encoding strategy within a stochastic optimal control framework by constructing control policies $\pi_{q_{j}}\left(x_{t}\right)=U^{q_{j}}=\left[u_{1}^{q_{j}},u_{2}^{q_{j}},...,u_{t_{end}}^{q_{j}}\right]$ for every target location that are optimal with respect to a cost function [18]—*j* = 1, 2 for the current example described in Fig 1B (right panel). A wide variety of models have been proposed in the literature about what cost functions the sensorimotor system might use to generate motor behavior (i.e., trajectories) that matches well experimental data. Such examples include the minimum jerk model [32], the minimum torque-change model [33], the minimum end-point variance planning model [34] and the extended LQG model [35]. In the current study, we used the minimum end-point variance planning as a cost function on the control system to generate single-target motor plans (i.e., policies). The optimal policy $\pi_{q_{j}}\left(x_{t}\right)$ for reaching to the target location *q*_*j*_ from the current state *x*_*t*_ is given by minimizing the following cost function:

$$\begin{array}{c} J_{j}(x,\pi_{q_{j}})={\left(x_{T_{j}}-Sq_{j}\right)}^{T}Q_{T_{j}}(x_{T_{j}}-Sq_{j})+\sum_{t=1}^{T_{j}-1}\pi_{q_{j}}{\left(x_{t}\right)}^{T}R\pi_{q_{j}}(x_{t}) \end{array}$$ (5)

where the first term describes the accuracy cost at the end of the movement and the second term the effort cost from the movement onset to the end of the movement. Also, *T*_*j*_ is the time-to-arrive to the target location *q*_*j*_ starting from the current state *x*_*t*_, and *S* is a square symmetric matrix (for more information see [18]). The main question is how to combine the individual optimal policies $\pi_{q_{j}}\left(x_{t}\right)$ into a single policy to be executed. We showed that optimal control policies associated with the alternative targets can be combined using a *relative desirability* function $rD(\pi_{q_{j}}\left(x_{t}\right))$ for each policy $\pi_{q_{j}}$ computed at the state *x*_*t*_ [18]. The relative desirability integrates information from disparate sources with different “currencies” (i.e., expected reward and action cost) into a single value in a manner that is both online and can be updated during action execution [18]. It is time-varying and depends on the current state of the trajectory, the target probability and the effort cost to implement the control policy from the current state. It reflects the probability of getting the highest outcome with the least effort if adopting the policy $\pi_{q_{j}}\left(x_{t}\right)$, Eq (6).

$$\begin{array}{c} rD\;(\pi_{q_{j}}\left(x_{t}\right))=P\;(Outcome_{\pi_{q_{j}}}\left(x_{t}\right)>Outcome_{\pi_{q_{i\neq j}}}\left(x_{t}\right))\;P\;(Cost_{\pi_{q_{j}}}\left(x_{t}\right)<Cost_{\pi_{q_{i\neq j}}}\left(x_{t}\right))+\xi_{t} \end{array}$$ (6)

where the first term of Eq (6) describes the probability that policy $\pi_{q_{j}}\left(x_{t}\right)$ will produce the highest outcome with respect to any other alternative, the second term is the probability that $\pi_{q_{j}}$ will consume the lowest effort with respect to any other alternative $\pi_{q_{i\neq j}}$, at a current state *x*_*t*_, and *ξ*_*t*_ is the error in the relative desirability estimation sampled from a Normal distribution $N(\mu_{\xi },\sigma_{\xi }^{2})$. Note that the effort cost is computed directly from the 2^*nd*^ term in cost function of Eq (5).

After computing the relative desirability values, the control policy *π*_*mix*_(*x*_*t*_) that will generate the reach trajectory (black discontinuous trace in Fig 1B right panel) is given as:

$$\begin{array}{c} \pi_{mix}\left(x_{t}\right)=\sum_{j=1}^{K}rD(\pi_{q_{j}}\left(x_{t}\right))\pi_{q_{j}}\left(x_{t}\right) \end{array}$$ (7)

where *K* is the total number of potential targets (*K* = 2 in the current example showing on the right panel in Fig 1B). The implementation of this theory within a neurodynamic framework is presented in the materials and methods section.

This computational analysis revealed that the main difference between the two encoding strategies is where the uncertainty is resolved. According to the single action strategy, the uncertainty about the current best action is resolved in the target space. The reaction time depends on how quickly the brain plans and executes a single motor plan strategy in the presence of goal location uncertainty. On the contrary, according to the motor encoding strategy, uncertainty is resolved in the action plan (i.e., policy) space. The reaction time depends on the competition between the action plans. The faster the competition is resolved, the shorter the reaction time. The current study aims to decipher what encoding strategy people adopt in reaching movements with multiple potential goals, in order to understand how the brain computes and utilizes uncertainty into motor behavior.

### 2.2 Behavioral paradigm

**Fig 2:**
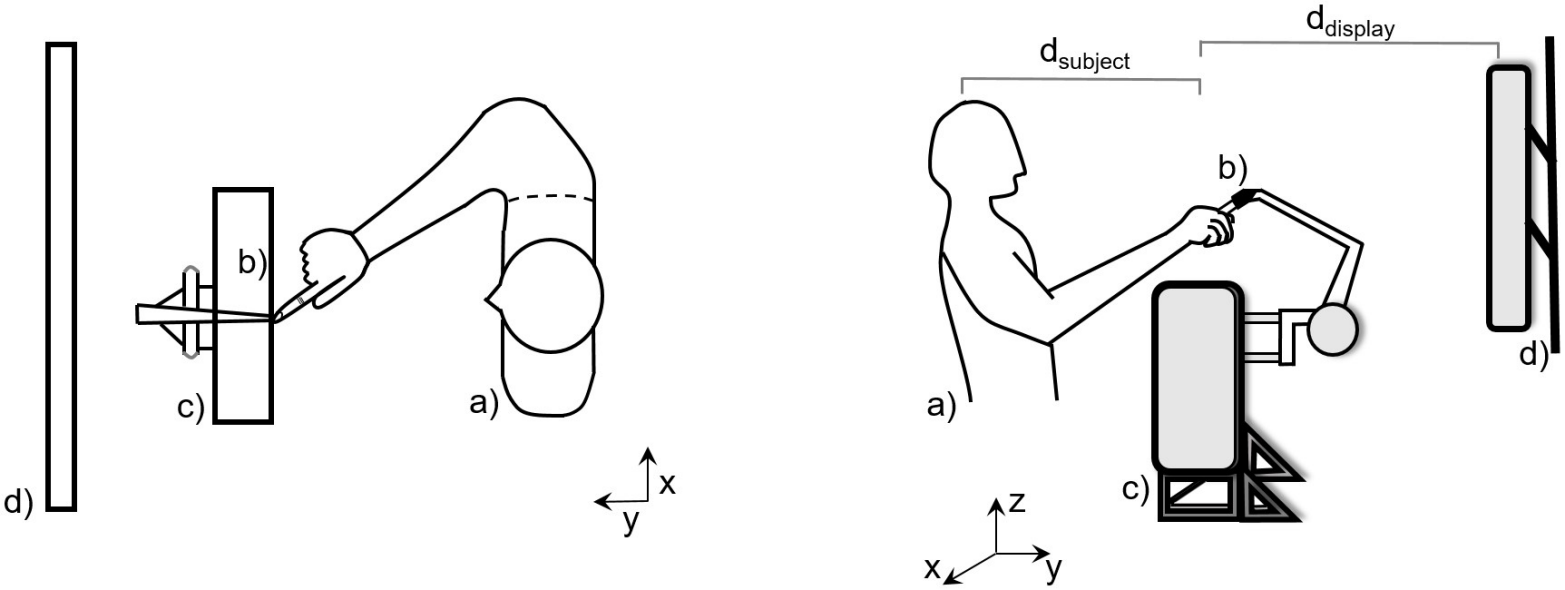
A graphical representation of the experimental setup from two perspectives. Participants (a) were seated directly in front of a Phantom haptic robot (c), with their index fingers inserted in a finger-tip adaptor (b) and their midline aligned with the center of an LCD monitor (d). Reaching movements took place in the *x* − *y* plane, +*y* being towards the screen and +*x* being towards the right hand side of the screen. The distance from the head of the individuals to the finger starting position along the *y* axis was about *d*_*subject*_ = 0.30 m and slightly varied across participants. The distance from the finger starting position to the screen display was *d*_*display*_ = 0.35 m.

**Fig 3:**
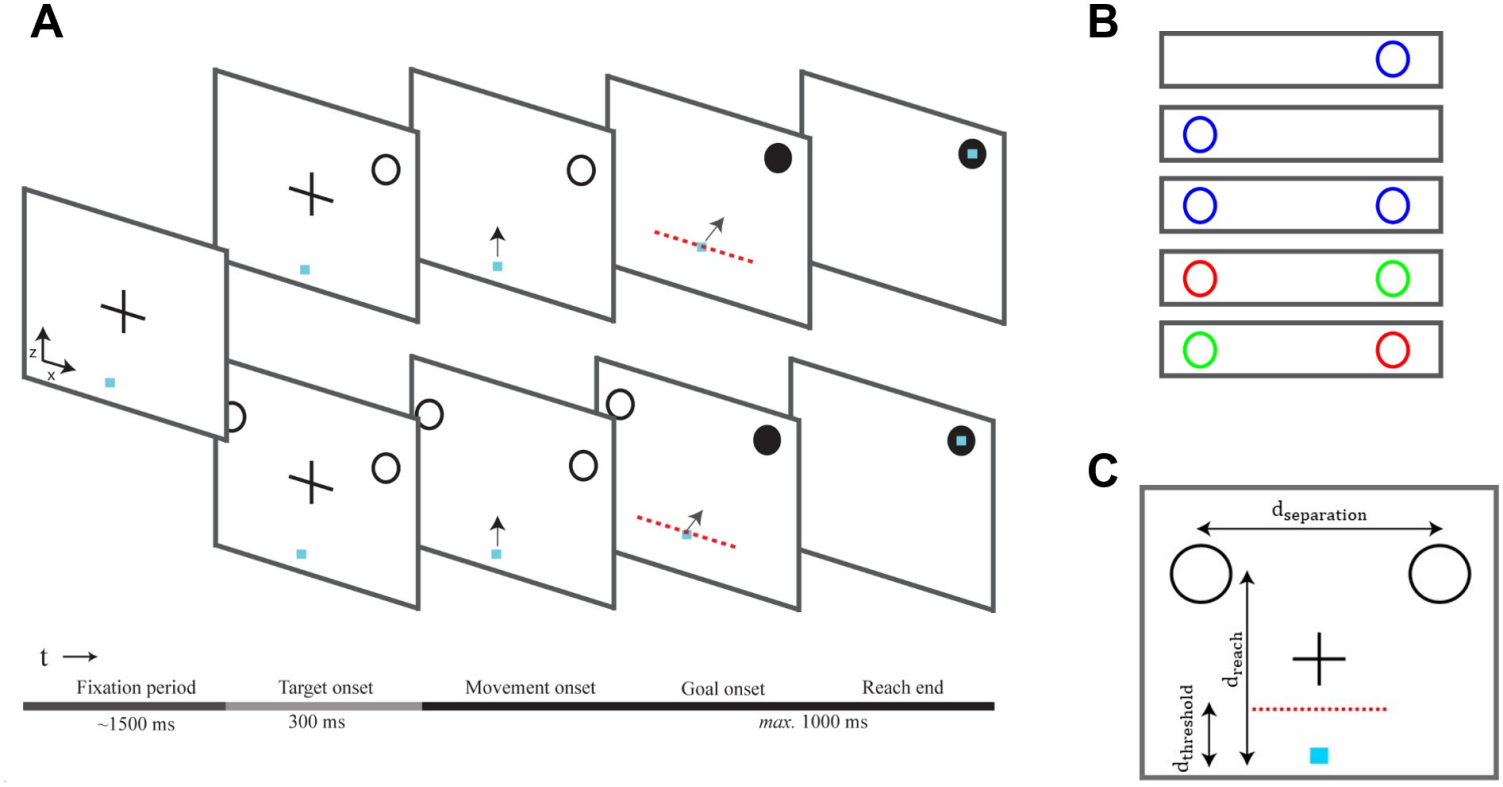
Task design and experimental paradigm. (**A**): A reaching trial started with a fixation cross presented on the center of the screen for about 1.5 s. Then, either a single or two unfilled cues were presented simultaneously in both visual fields. After 300 ms the central fixation cross was extinguished (“go-signal”), and the participants had to perform a rapid reaching movement towards the target(s) within 1 s. Once the reach trajectory crossed a trigger threshold (red discontinuous line), one of the cues (or the single cue) was filled-in black indicating the actual goal location. Responses before the go-signal or reaches that exceeded the maximum movement time (1 *s*) were aborted and not used for further analysis. (**B**): The color of the cues in the dual-target trials indicated the target probabilities—blue cues corresponded to equiprobable targets, whereas green and red cues corresponded to targets with 80% and 20% probability, respectively. Single cues always had blue color. (**C**): The distance between the origin and the midpoint of the two cues was *d*_*reach*_ = 0.2 *m*. The distance between the two targets was *d*_*separation*_ = 0.30 *m*. The trigger threshold—i.e., distance between the origin and the location that the actual goal location was revealed—was set to *d*_*threshold*_ = 0.05 *m*.

A schematic representation of the experimental setup is shown in Fig 2. Participants were instructed to perform rapid reaches using a robotic manipulandum under a “reach-before-you-know” paradigm [6, 36] in which either one (single-target trials) or two (dual-target trials) potential targets presented simultaneously in opposite hemifields. For dual-target trials, the cues appeared symmetric around the vertical axis of the screen. By varying the number of potential targets and their probabilities, we study how individuals plan and execute actions under goal location uncertainty. In the **equiprobable** session, each trial started with participants fixating on a central cross, followed by the presentation of one or two unfilled blue circles in the screen Fig 3A. When the fixation cue was extinguished, an auditory cue signaled the individuals to initiate their responses. Once the reaching movement exceeded a threshold, one of the targets filled-in black indicating the actual goal location. The **unequiprobable** session was similar to equiprobable except for the dual-target trials, in which one of the potential targets was always assigned with higher probability (0.8) than the alternative one (0.2). The targets with the high and low probabilities were indicated by unfilled green and red cues, respectively. In single-target trials (i.e., target probability = 1) which were randomly interleaved with the dual-target trials in both sessions, a single unfilled blue cue was presented in the left or the right hemifield. The set of target configurations is shown in Fig 3B. Participants achieved an overall success rate around 93% and their performance was similar between the two sessions (93% and 90% respectively).

### 2.3 Initial approach direction varies with target probability

**Fig 4:**
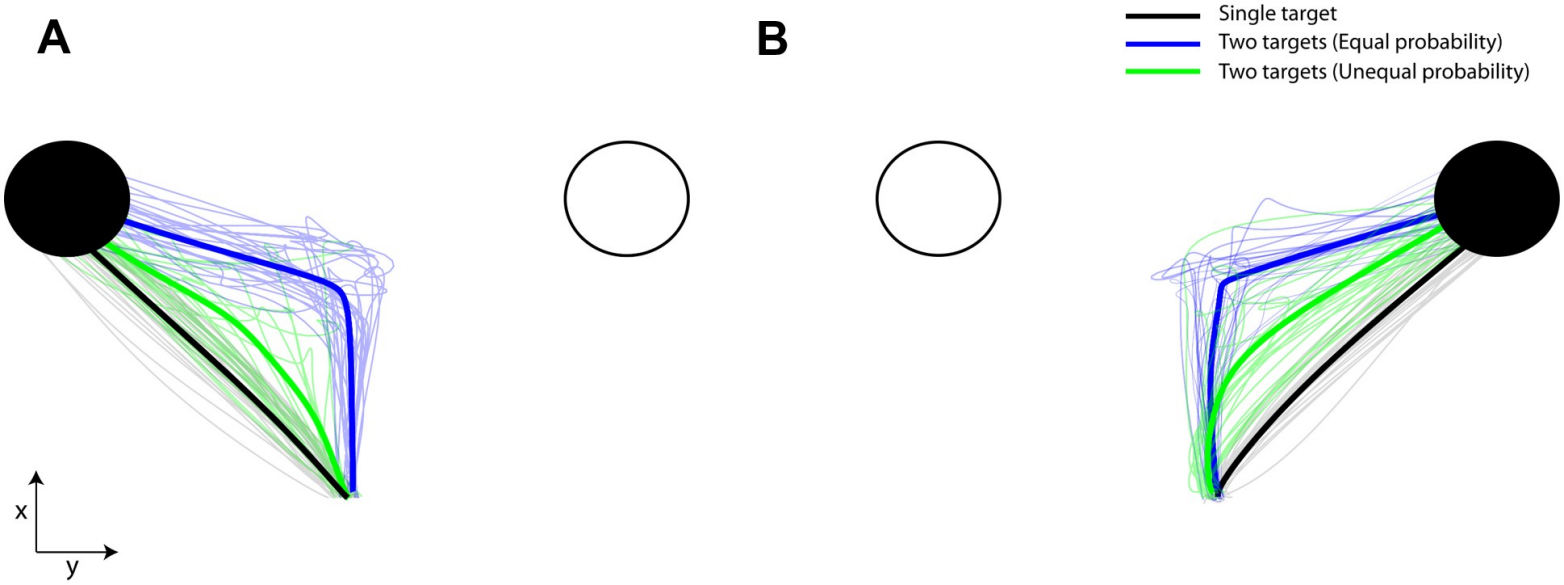
Reach trajectories for different target probabilities. (**A**): Representative single-trial trajectories (thin traces) and the corresponding average trajectories (thick tracres) from single- (black trace) and two-target trials with equal (blue trace) and unequal (green trace) probabilities, when the actual goal was located in the left hemifield. (**B**): Similar to A but for actual goal located in the right hemifield. Target probability influences the reach trajectories. When people were certain about the goal location, reaches were aimed directly to the target. When they were uncertain, reaches were launched to an intermediate location between the targets and then corrected in-flight to the cued target location. The spatially averaged behavior was biased towards the likely target.

**Fig 5:**
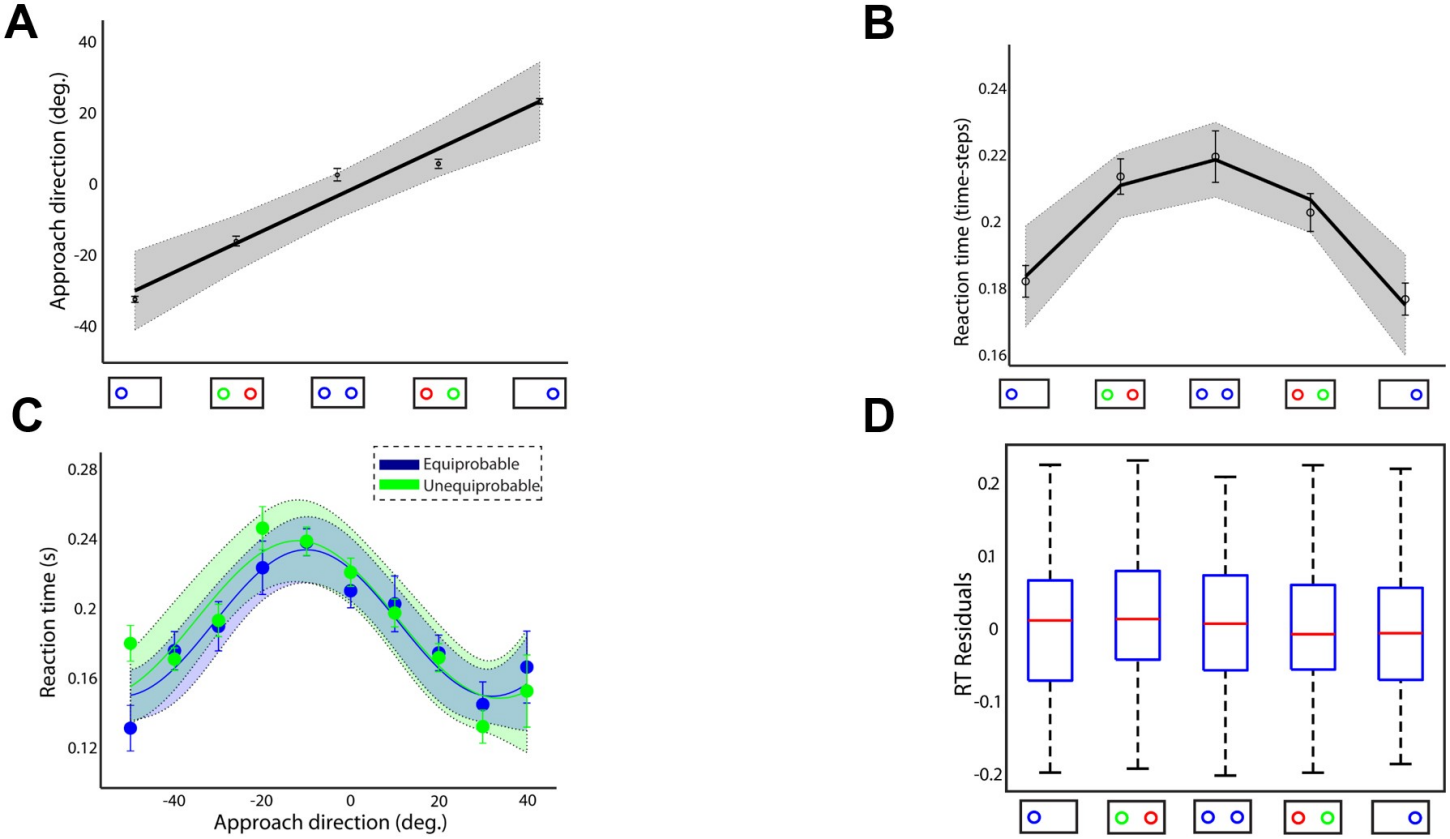
Approach direction and reaction time. (**A**): Approach direction and (**B**) reaction time across participants, number of targets and probabilities. Positive and negative approach directions correspond to reaches launched closer to the right and left target, respectively. Approach directions around 0° correspond to reaches aimed towards the midline location between the two targets. (**C**): Reaction time as a function of the approach direction in equiprobable (blue trace) and unequiprobable (green trace) sessions (**D**): Boxplots of the RT residuals (i.e., difference between actual and predicted RTs) for the 5 different target probabilities. Error bars correspond to standard error (SE), solid lines show the regression fitting (linear in panel A, quadratic in panel B and cosine in panel C) and the colored shadow areas illustrate the confidence interval of the regression results.

Target probability is well known to have a strong effect on reaches, where the initial movement trajectory is aimed between targets. This motor behavior, which has been extensively reported before [6, 9, 12, 16], indicates that the approach direction of the initial reaches varies with the target probability, a finding we replicated. Representative single-trial trajectories (thin traces) from different target probabilities and the corresponding average trajectories (thick traces) for goal located in the left and right hemifield are illustrated in Fig 4A and 4B, respectively. When the goal location was known prior to movement onset, reaches were made directly to the goal target (black traces). Otherwise, reaches were aimed to an intermediate position between the potential goal locations (blue traces). The “spatial averaging” reach trajectories were reliably biased towards the side of space with the most likely target (green traces). Hence, individuals did not pre-select one of the potential targets prior to movement onset. Instead, they delayed their decisions by moving towards an intermediate location to collect more information before taking the final action. We compared the approach direction across participants, number of targets and probabilities and found that it is directly correlated with the target probability (best fit linear regression model; R-square = 0.967, p-value = 0.00258 of the linear coefficient) Fig 5A. Our findings suggest that when people are faced with multiple competing options, they both delay their decision and move towards an intermediate location between the targets, a strategy consistent with increasing chances of collecting more information before making a choice.

### 2.4 Reaction time depends on the initial approach direction

The dual effects of target probability on reach trajectory and timing suggest that uncertainty is incorporated into both acting (trajectory generation) and planning processes. Intuitively, it is reasonable that target probability influences action planning to delay initiating action when uncertain about the best option. This predicts reaction time (RT) would be a direct function of target probability. On average, this prediction is validated as illustrated in Fig 5B for single-target trials, two-target trials with equal probability and two-target trials with unequal probability, with RT averaged across participants (best fit quadratic regression model; R-square = 0.98, p-value = 0.0197 of the quadratic coefficient). However, the initial approach direction is also correlated with the target probability, raising the question whether the association between RT and target probability is related to the target probability itself or to the initial approach direction. A trial-by-trial analysis showed that the effect on initiation timing was indirect and actually mediated by a latent variable influencing both RT and approach direction of a trajectory. By plotting RT vs. approach direction separately for the two sessions, we found that changes in RT are independent of target probability and accounted for by approach direction. Fig 5C shows RT as a function of the initial approach direction across all participants and trials separately for the two sessions. Importantly, RT increases with reaches to intermediate locations regardless of the target probability (approach direction was sorted into 10 consecutive bins and then mean RT was computed in each bin; best fitting cosine curve model; R-square > 0.878, p-value < 0.0038 in both sessions). To ensure that this effect was not due to some inherent constraints induced by the experimental setup—i.e., reaches aimed to the center of the screen have longer RTs than reaches aimed to peripheral targets—we varied the target location between 0.10 m to 0.20 m from the midline in the equiprobable session and computed the RT in the single-target trials. No significant association was found between target location and RT (p-value > 0.197 of the regression coefficients for linear and curvilinear regression analysis, S4 Fig).

To further explore the association between approach direction and RT, we tested how much of the variation in RT is explained by approach direction and how much is explained by target probability. To do so, we modeled the relationship between approach direction and RT in the equiprobable condition by fitting a nonlinear regression model across all trials (cosine curve fitting across all trials and participants, R-square = 0.138, p-value <10^−28^) and then we used this model to predict RT from any given approach direction across all different target probabilities in both equiprobable and unequiprobable conditions. Fig 5D illustrates the boxplots of the RT residuals (i.e., difference between actual RTs and predicted RTs) for all different probability values. The results showed that there are no significant changes on RT residuals across target probabilities indicating that RT is independent of the target probability (one-way ANOVA F(4,1466) = 1.96, p = 0.097). This finding suggests that the association between RT and target probability illustrated in Fig 5B is not directly related to the target probability itself. Instead, it is related to the approach direction. For instance, reaches exhibit longer RTs in two-target trials with equal probability than in single-target trials, because the first condition involves more reaches to intermediate locations than the latter one that involves mainly direct reaches to the target locations. Overall, our findings suggest that changes in RT are independent of target probability, and the approach direction and RT are driven by trial-by-trial variations in a latent variable, which we identify with *relative desirability* as we describe in the following section.

### 2.5 Action selection and reaction time emerge through action competition

**Fig 6:**
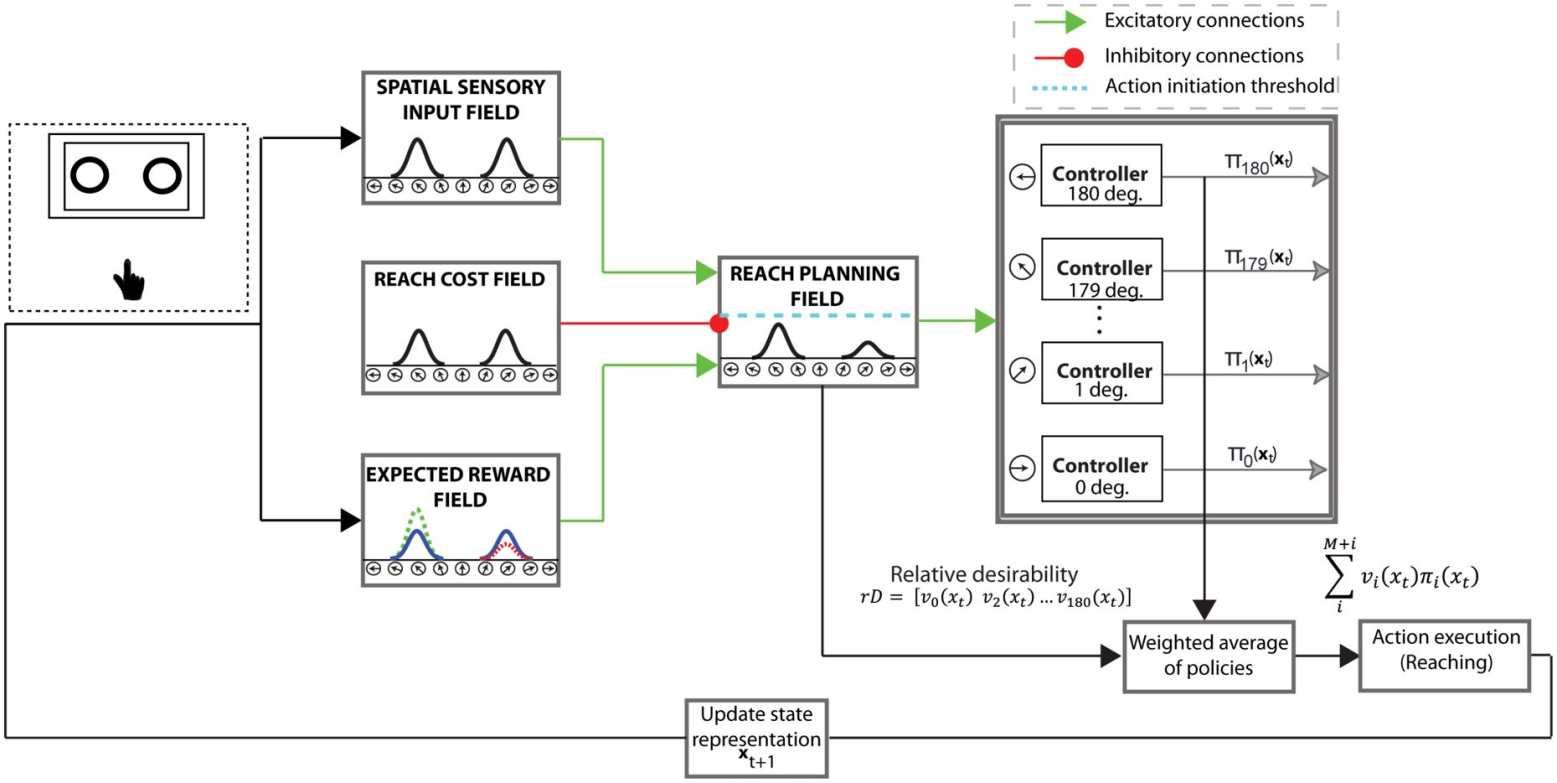
Model architecture of the “reach-before-you-know” task. The neural fields consist of 181 neurons and their spatial dimension spans the semi-circular space between 0° and 180°. Each neuron in the reach planning field is connected with a stochastic optimal control system. Once the activity of a neuron exceeds a threshold *γ*, the corresponding controller generates a sequence of reach actions towards the preferred direction of the neuron. The reach planning field receives excitatory inputs from the spatial sensory input field that encodes the angular representation of the potential targets, and the expected outcome field that encodes the expected outcome of the competing targets (blue, red and green Gaussian distributions correspond to cues with 0.5, 0.2 and 0.8 target probability, respectively). It also receives inhibitory inputs from the reach cost field that encodes the effort required to implement the available sequences of actions—i.e., move to a particular direction from the current state. The normalized activity of the reach planning field encodes the “desirability” of the *M* available sequences of actions (i.e., neurons with activation level above the threshold *γ*) at a given time and state and acts as a weighting factor on each individual sequence of actions. Because the relative desirability is time- and state- dependent, a range of behavior from weighted averaging (i.e., spatial averaging trajectories) to winner-take-all (i.e., direct reaches to one of the cues) is generated.

Our results require a decision computation that would produce joint changes in trajectory and RT as a function of trial-by-trial fluctuations in a latent variable. We developed a theory [17, 18] that predicts exactly these effects using *relative desirability* as the latent variable. The relative desirability is a dynamic variable that describes how desirable is to perform an action with respect to the alternatives at a given time and state. It is related to choice uncertainty, since if one action has higher desirability value and outperforms the alternatives, the action competition is low and hence there is less uncertainty as to which option to act up. In this theory, action decisions are made through a continuous competition of parallel prepared actions (motor encoding strategy) by dynamically integrating all sources of information about the quality of the alternative options. The neurodynamic implementation of this theory for a dual-target trial is presented in Fig 6. The framework consists of a set of dynamic neural fields (DNFs), which mimic the neural processes underlying spatial sensory input, expected outcome, reach cost (i.e., effort) and reach planning [17]. Each DNF simulates the dynamic evolution of firing rate activity within a neuronal population. The functional properties of each DNF are determined by the lateral interactions within the field and the connections with other fields [37, 38].

The “reach planning” field employs a neuronal population code over 181 potential movement directions to plan motor actions towards these directions. It receives one-to-one excitatory inputs from the “spatial sensory input” field that encodes the angular representation of the targets and the “expected outcome” field that represents the expected outcome of aiming to a particular direction. Each neuron in the reach planning field is projected to a stochastic optimal control system. Once the activity of a reach neuron *i* exceeds a threshold *γ* at the current state *x*_*t*_, the corresponding controller initiates an optimal policy *π*_*i*_(*x*_*t*_) to move the “hand” towards the preferred direction of that neuron (see Materials and methods section for more details). The reach planning field receives also inhibitory inputs from the “reach cost” field that encodes the effort required to implement each policy *π*(*x*_*t*_) at the current state. The normalized activity of the reach planning field represents the *desirability* of the motor actions at any time and state, and acts as a weighting factor on them. It reflects how “desirable” it is to move to a particular direction with respect to the alternatives. Because desirability is time- and state- dependent, the weighted mixture of individual actions automatically produces a range of behavior, from direct reaching movement to weighted averaging.

**Fig 7:**
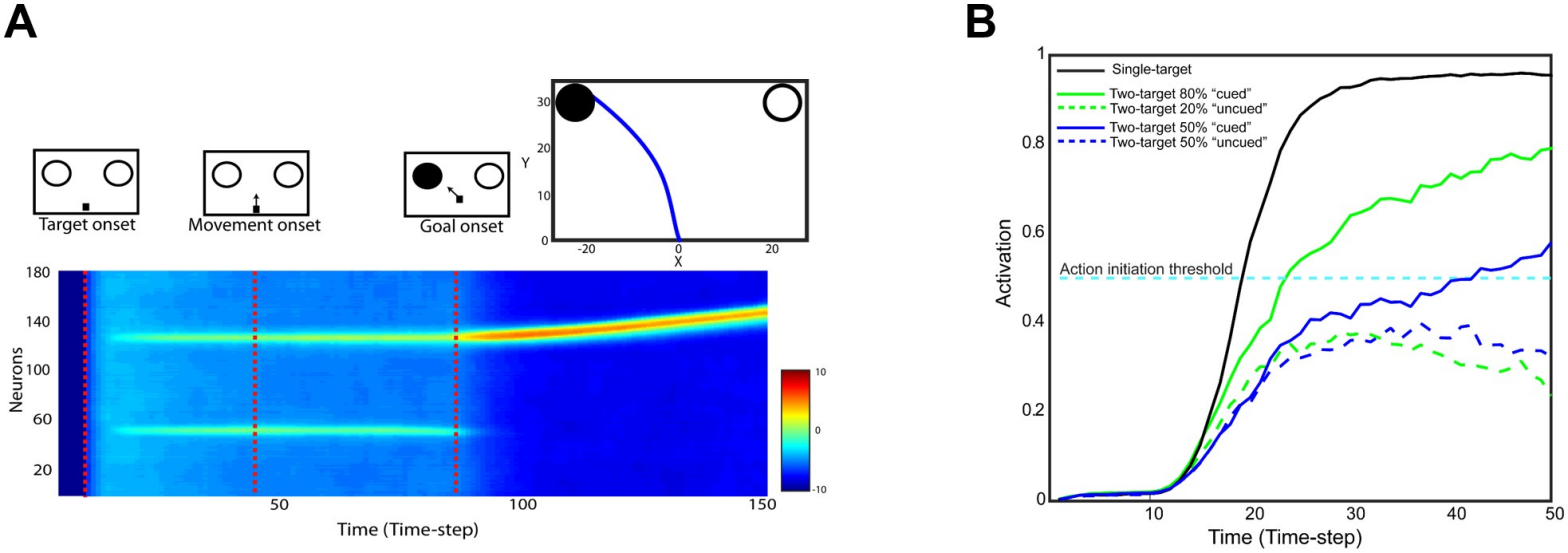
Simulated neural activity and reach behavior. (**A**): A representative example of the simulated model activity as a function of time in the reach planning field for a dual-target trial with the actual goal located in the left visual field. The red discontinuous lines indicate the target onset, the movement onset, and the goal onset. The corresponding reach trajectory is shown in the upper inset. (**B**): Simulated activity of two planning neurons centered at the location of the cued (continuous traces) and the uncued (discontinuous traces) target, from a representative single-target trial (black trace) and two dual-target trials with equal (blue traces) and unequal (green trace) probabilities. A reach movement is initiated when the activity of one of the neurons exceeds the response threshold (gray discontinuous trace). When only a single target is presented, the neuronal activity ramps up quickly to the response threshold resulting in faster reactions and direct reaches to the target. However, when two targets are simultaneously presented, the neurons compete for selection through inhibitory interactions resulting often in slower reaction times and spatially averaged movements. If one of the alternatives is assigned with higher probability, the competition is biased to the likely target leading to faster responses.

Fig 7A illustrates the activity of the planning field as a function of time for a representative dual-target trial with equiprobable targets. Initially, the field activity is in the resting state. After targets onset, two neuronal populations selective for the targets are formed and compete through mutual inhibitory interactions, while integrating information about the target probability and action cost to bias the competition. Once the activity of one of them exceeds a response threshold, the corresponding target is selected and a reaching movement is initiated. Frequently, the neuronal activity of the unselected target is not completely suppressed before movement onset, resulting in reaches towards intermediate locations between the targets (top inset in Fig 7A). After the movement onset, the two neuronal ensembles retain activity and compete against each other until the goal onset.

To get better insight on the model computations consider two neurons, one from each population, centered at the target locations. Fig 7B depicts the activity of each neuron (i.e., which reflects its current desirability value) as function of time for a dual-target trial with equal (blue traces) and unequal (green traces) target probability. The neuron that exceeds the response threshold first (continuous traces) dictates the reaction time and the selected target. Intuitively, if the race between the neurons is a close call (blue traces), the net evidence supporting that one action is more desirable than the alternative is weak, resulting in higher uncertainty and intermediate movement directions. On the other hand, if the race was a landslide (green traces), one alternative outperforms the other resulting in lower uncertainty and direct movements to one of the targets. Going back to the population analysis, the “winning” population determines the reaction time and the selected target, whereas the “losing” one contributes to the computation of the momentary uncertainty about the current best action—i.e., the larger the difference between the desirability values of the alternative actions the lower the uncertainty about the current best action. Note that in the absence of action competition (i.e., single-target trials), the activity of the neuron exceeds the response threshold faster than when two actions compete for selection (black trace). Hence, reaches have shorter reaction time and aim directly to the goal location. Overall, the theory is analogous to the normative race models in perceptual decisions in which two accumulators integrate sensory evidence in favor of two alternative options [23, 39]. The accumulator that reaches its upper bound faster dictates the reaction time and the choice, whereas the losing accumulator contributes to the computation of uncertainty that the choice is correct (balance-of-evidence hypothesis [40]).

**Fig 8:**
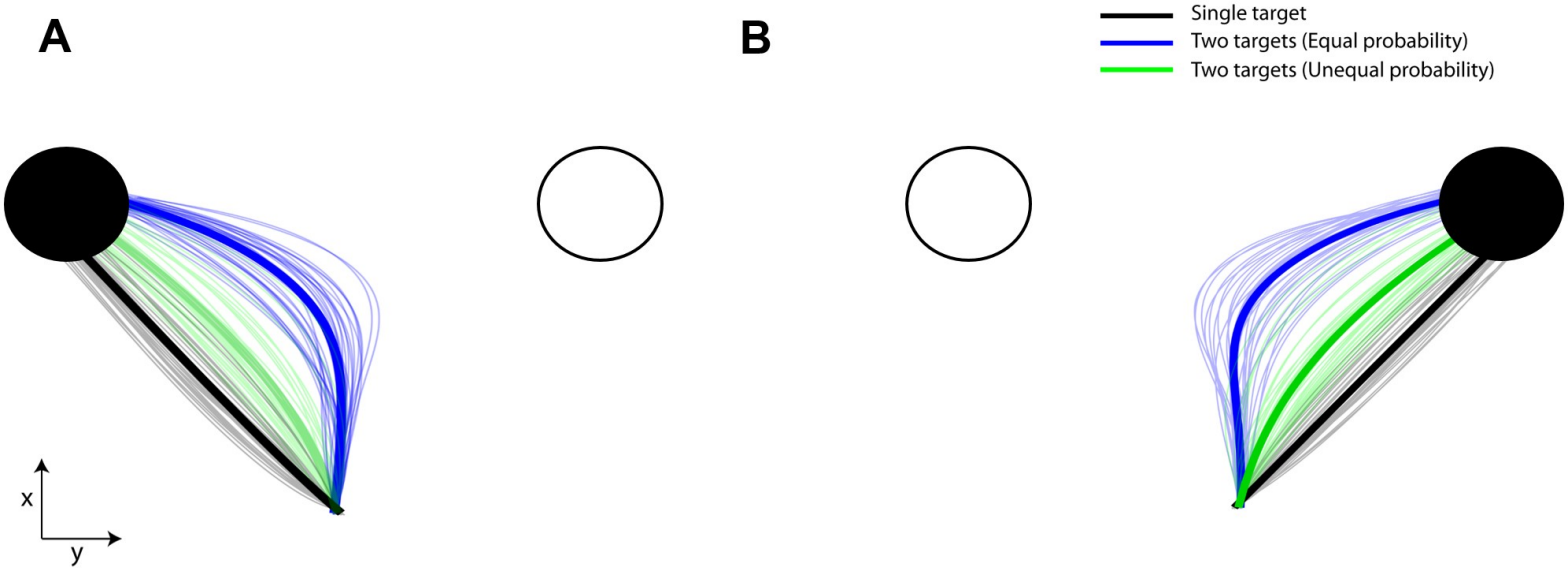
Simulated reach trajectories for different target probabilities. (**A**): Representative single-trial simulated reach trajectories (thin traces) and the corresponding average trajectories (thick tracres) from single- (black trace) and two-target trials with equal (blue trace) and unequal (green trace) probabilities, when the actual goal located in the left hemifield. (**B**): Similar to A but for actual goal located in the right hemifield. Consistent with the human behavior, initial simulated movements are launched towards an intermediate (i.e., averaged) spatial location (blue traces), when the actual goal is unknown prior to movement onset. The spatial behavior is biased by the target probability (green traces). When the actual goal is known before movement initiation, reaches are aimed directly to the target location (gray traces).

**Fig 9:**
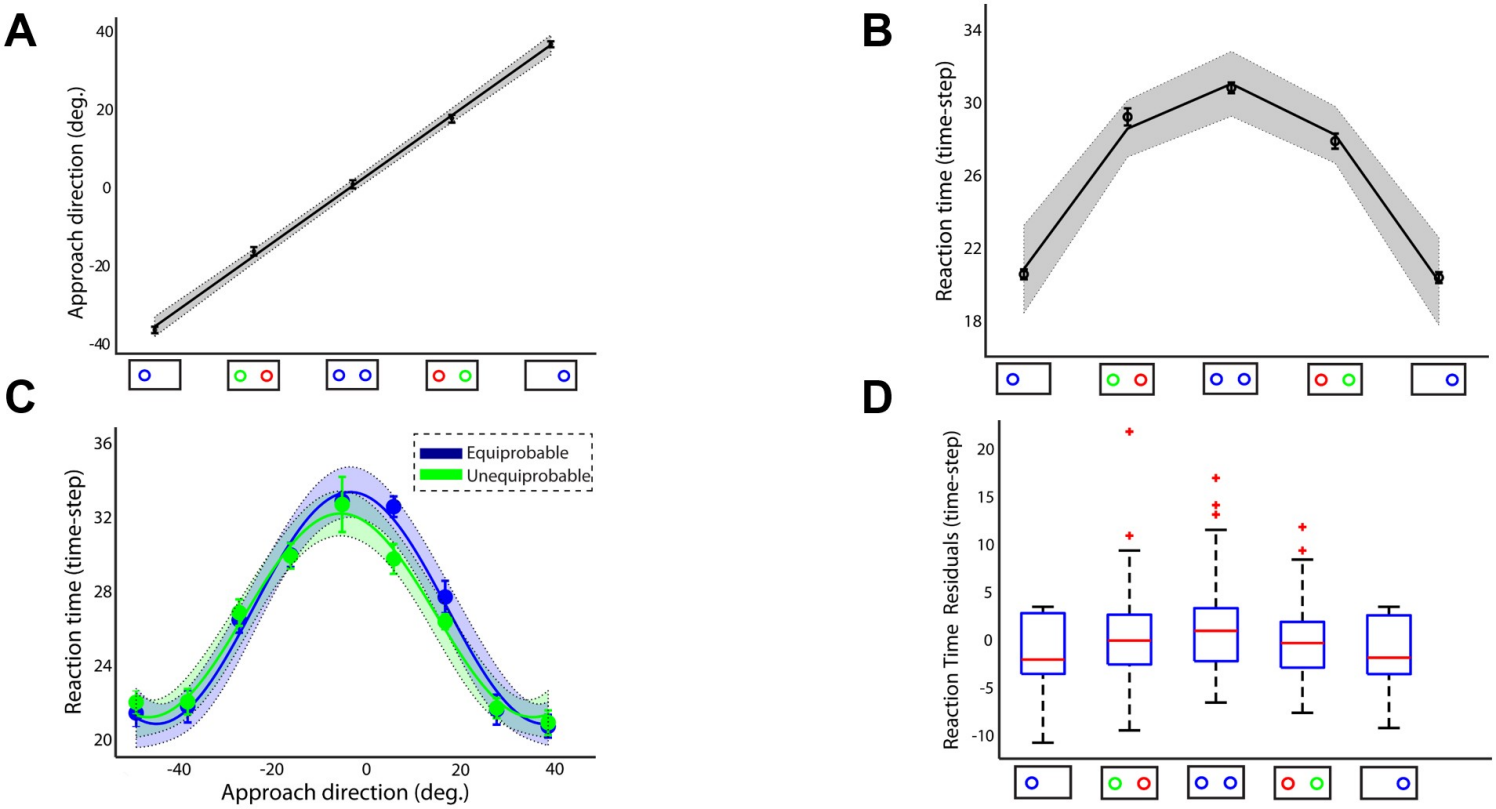
Approach direction and reaction time of the simulated reaches for motor encoding strategy. (**A**) Approach direction and (**B**) reaction time of the simulated reaches across number of targets and probabilities. (**C**): Reaction time as a function of the approach direction in the simulated equiprobable (blue trace) and unequiprobable (green trace) sessions. (**D**): Boxplots of the RT residuals (i.e., difference between actual and predicted RTs) for the 5 different target probabilities. Error bars correspond to standard error (SE), solid lines show the regression fitting (linear in panel A, quadratic in panel B and cosine in panel C) and the colored shadow areas illustrate the confidence interval of the regression results.

We simulated the two equiprobable and unequiprobable sessions within the computational theory (350 trials for each session, 2 x 350 = 700 trials total), using the parameter values presented in S1 Table. Representative single-trial trajectories (thin traces) for different target probabilities and the corresponding average trajectories (thick traces) for goal located in the left and right hemifield are illustrated in Fig 8A and 8B, respectively. The model predicts that intermediate movements are launched (blue traces) when the goal location is unknown, and are biased towards the target with the highest probability to be cued for action (green traces). When the actual goal is known prior to movement initiation, reaches are aimed directly to the goal location (black traces). Therefore, consistent with the human behavior, the target probability is correlated with the approach direction Fig 9A (best fit linear regression model: R-square = 0.999, p-value = 0.000013 of the linear coefficient) and RT Fig 9B (best fit quadratic regression model: R-square = 0.993, p-value = 0.007 of the quadratic coefficient). We also tested trial-by-trial association between RT and approach direction and found the same independence from target probability, Fig 9C (approach direction was sorted into 9 consecutive bins and then mean RT was computed in each bin; best fit cosine curve model in both sessions: R-square = 0.984, p-value = 0.00006). In particular, simulated reaches aimed towards an intermediate location between the potential targets had longer RT than reaches launched closer to one of the competing options regardless of the target probability. This is explained by the inhibitory interaction between the neuronal ensembles that slows down the reach onset and leads to spatial averaging movements, if the population of the unselected action is not completely suppressed at the movement initiation. Finally, we performed the same analysis with the human experiment to explore whether the association between RT and approach direction is mediated by the target probability. In particular, we modeled the association between RT and approach direction in the simulated equiprobable session across all trials (cosine curve fitting across all trials, R-square = 0.60, p-value = 0.00001) and then we used this model to predict RT from any give approach direction across all different target probabilities in both equiprobable and unequiprobable sessions. Fig 9D shows the boxplots of the RT residuals (i.e., the difference between predicted RT and actual RT) for different target probabilities. The results showed similar trend with the human data—i.e., target probability does not have a strong effect on the RT—but contrary to the human data, the RT residuals of the simulated reaches exhibit small, but statistically significant, changes with target probability (one-way ANOVA, F(4,595) = 6.82, p < 0.00002). A post-hoc multiple comparison analysis showed that RT residuals were statistically significant different (p < 0.05) only between single target trials and two-target trials with equal probability. One potential explanation of the difference between the human and the simulated RT residuals is that simulated reaches do not take into account the history of previous trials. Instead, it assumes that every trial is independent on the previous ones. On the other hand, in the human experiment, two-target trials were interleaved with single target trials, and therefore, even when participants were instructed to perform direct reaches to single targets, their reaching behavior might be influenced from previous two-target trials. The lack of this *sequential effect* in the computational model can explain the small, but statistically significant, difference on the RT residuals between single target trials and two-target trials with equally probable targets. Overall, the model predicts that the degree of action competition influences the initial approach direction and reaction time of the reaching movements. This suggests that action selection, reaction time and uncertainty about the best current action emerge through a common mechanism of desirability-driven competition between parallel prepared actions.

### 2.6 Reach behavior does not reflect a single motor plan strategy

So far, our findings are in favor of the motor encoding strategy. However, spatial averaging behavior could be also explained within the family of single motor plan strategies, such as visual averaging encoding or performance optimization strategies. From a computational perspective, these strategies have the advantage that the brain does not need to allocate resources to forming motor representations of all potential targets [41]. However, the main critique against the performance optimization strategy is that it does not actually address how such optimized movements may themselves be computed or explain the neural representation on which they are based [30]. Behaviorally, single motor plan strategies can predict some components of motor behavior in rapid reaching movements, such as the initial approach direction—e.g., reaches are aimed towards a visual average location between the targets, weighted by the target probabilities. It can also be argued that they can explain the reaction time as a function of the target probability presented in Fig 5B, if we hypothesize that participants performed frequently direct reaching movements to the high probability target while ignoring the low probability target. Therefore, the average reaction time on the unequiprobable trials would be shorter than the equiprobable trials. The last component of motor behavior that remains to be tested is whether a single motor plan strategy, without an action competition mechanism, can explain the association between reaction time and approach direction.

**Fig 10:**
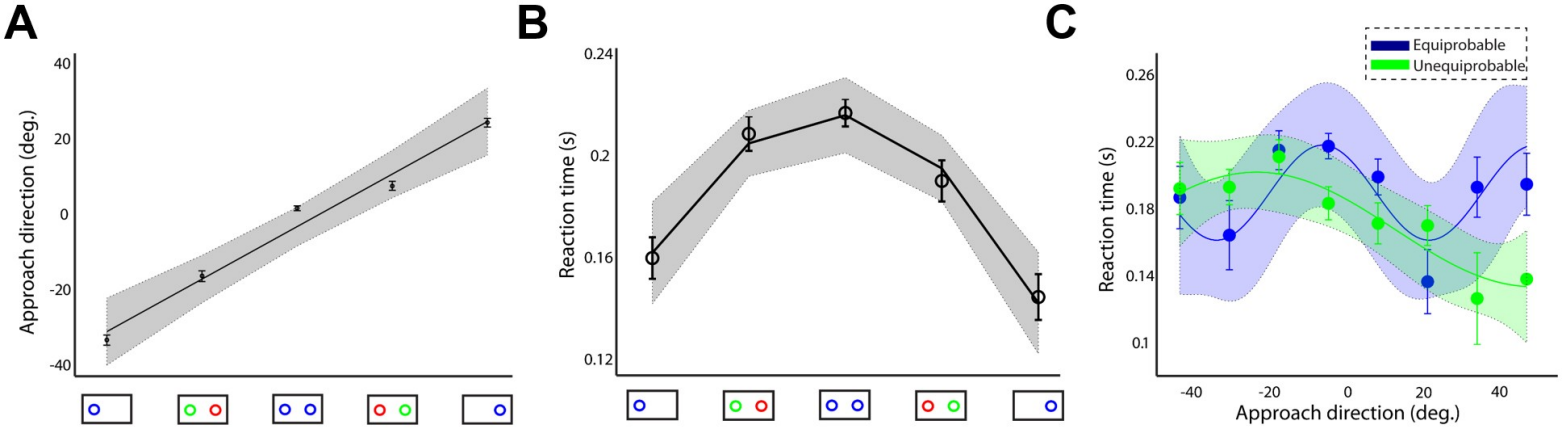
Approach direction and reaction time of the simulated reaches for a hypothetical single motor plan strategy. (**A**): Approach direction and (**B**) reaction time of the simulated reaches across number of targets and probabilities. (**C**): Reaction time as a function of the approach direction in the simulated equiprobable (blue trace) and unequiprobable (green trace) sessions. Error bars correspond to standard error (SE), solid lines show the regression fitting (linear in panel A, quadratic in panel B and cosine in panel C) and the colored shadow areas illustrate the confidence interval of the regression results. The single motor plan strategy fails to predict human behavior in motor decisions with competing targets.

To do so, let’s see what would happen if reaching movements were generated by a mechanism that does not include action competition. Instead, participants first decided where to move and then generated a single action towards this direction. In this case, RT would depend on the time that it takes to specify the visual target location and the time to plan a reaching movement towards this target location. To simulate this hypothesis, we sampled RT and approach direction from a Normal distribution with parameters the mean and the standard deviation of the reaching movements in each condition (i.e., target probability). For instance, in the two target trials with equal probability, if $M_{Dir_{50-50}}$ and $SD_{Dir_{50-50}}$ are the mean and the standard deviation of the participants’ approach direction in this condition, we generated the approach direction from a Normal distribution $N∼(M_{Dir_{50-50}},SD_{Dir_{50-50}})$. Similarly, reaction time is generated by a Normal distribution $N∼(M_{RT_{50-50}},SD_{RT_{50-50}})$, where $M_{RT_{50-50}}$ and $SD_{RT_{50-50}}$ are the mean and the standard deviation of the participants’ RT in the two target trials with equal probability. Although the lack of action competition can predict the correlation between approach direction and target probability Fig 10A (linear regression model; R-square = 0.98, p-value = 0.00118), as well as the association between RT and target probability Fig 10B (quadratic regression model; R-square = 0.987, p-value = 0.0126), it fails to predict that RT varies with the approach direction regardless of the target probability Fig 10C (cosine curve fitting; R-square = 0.656, p-value = 0.194 in the equiprobable session and R-square = 0.885, p-value = 0.024) as we found in the human data analysis. In other words, reaches that are aimed towards intermediate locations have not always longer RTs than reaches that aimed towards the target locations. Therefore, single motor plan strategies, in which people first decide where to move and then generate an action to implement their choice, cannot account for motor behavior in reaching movements with multiple competing targets, since they do not have a mechanism to predict the association between reaction time and approach direction.

## 3 Discussion

### 3.1 General

Uncertainty is ubiquitous in our interactions with the external world, and motor decisions regularly must be made in the face of it. Having to decide between competing options, there is often uncertainty that reflects our belief that a particular action is *better*, in the sense it is more likely correct or has a higher expected outcome than its alternatives. Over the past years, many studies have looked at how uncertainty emerges in decision making [23, 42–50]. Additionally, normative models, which include drift diffusion, evidence-accumulation, and race models [51–56], have been extended to understand the computations underlying choice uncertainty [23, 49]. Although parsimonious, most of the previous experimental studies are highly restricted and limited to perceptual choices made solely on the basis of the accumulation of sensory evidence and before individuals perform an action. They have primarily focused on the “decide-then-act” paradigms, in which individuals are completely motionless during deliberation, and generate an action only after committing to their final choice—although recent studies modeled evidence accumulation that continues after movement onset to account for “change-of-mind” that occurs during action execution [48, 49, 57]. In the “decide-then-act” studies, uncertainty is construed as reflecting the effective amount of sensory evidence at decision time, which is not adequate to account for choice uncertainty in motor decisions that are made while acting. For instance, sequential decisions within the context of foraging tasks involve continuous choices about which targets to act on and in what order [58]. In these tasks, target selection is made while acting and is often shaped by a balance between rewards and effort costs. Similarly, in a recent study people had to track a moving target, and occasionally, they were presented with a new target to which they could freely choose to switch at any time [59]. Decisions had to be made while acting by evaluating the advantage of switching an action or continue tracking the same target. Other studies explored the mechanisms of motor decisions by designing “go-before-you-know” paradigms, in which individuals had to initiate movements towards multiple potential targets, without knowing the actual goal location [6, 7, 10, 11]. In all these experimental paradigms, decisions evolve in dynamic and complex environments, in which the value and the availability of the options can change with time and previous actions, entangling decision process with action selection process. Here, individuals have to decide while acting among competing actions based on a variety of decisions variables, such as reward, effort, prior history, bias, etc. In these studies, uncertainty should be state- and time-dependent and reflect all the decision variables that affect our belief that an action is more *desirable* than the alternatives.

In the current study, we adopted this enriched view to explore how goal location uncertainty is encoded and resolved in motor decisions required during reaching to multiple potential goals. We hypothesized that the locus of uncertainty is over the set of single-target motor plans, which are generated concurrently and compete for selection. To test this hypothesis, we designed a “reach-before-you-know” experiment in which individuals were instructed to perform rapid reaches to one or two potential targets presented simultaneously in both hemifields. To elucidate the computations underlying uncertainty, we modeled the task within a recently developed computational theory [17, 18]. It is based on the idea that decisions are made through a continuous competition between neuronal populations that plan individual actions to the available goals, while dynamically integrating information into a common currency—named relative desirability—to bias the competition. The desirability reflects the belief about the quality of the action and acts as weighted factor on each individual action. The neuronal population that first exceeds a response threshold dictates the reaction time and the selected target. The competing population that did not exceed the threshold contributes to the computation of the momentary degree of uncertainty about the current best action; the closer the “losing” population to the threshold the higher the uncertainty about the selected action. When the activity of the losing population is not completely suppressed, reaches are aimed towards an intermediate location between the targets. Because desirability is time- and state-dependent, the momentary degree of uncertainty about the best current action can change in-flight in the presence of new incoming information affecting motor behavior and leading to “change-of-mind”.

The model predicts a direct association between target probability with initial approach direction and reaction time. When both targets are equally probable, the competition between the two populations is frequently a close call, which means that the net evidence supporting the selected action is weak and hence the uncertainty about the current best action is high. This results in slower reaction times and spatially averaged movements to an intermediate location between the potential goals. On the contrary, when one of the targets is assigned with higher probability, the competition is biased to the likely target. In this case, the net evidence supporting the selected action is strong and therefore the uncertainty about the current best action is low. This results in faster reaction times and more direct reaches to the selected target. Interestingly, the model predicts that the longer it takes to initiate an action, the more likely it is that the losing population will still be active at the movement onset, resulting in higher uncertainty about the current best action and spatially averaged movements. Hence, reaction time and approach direction are not fully mediated by the target probability, but they are influenced by the relative desirability (and consequently the choice uncertainty) of the alternative actions.

Consistent with the model predictions, individuals adopted a spatial averaging behavior to compensate for the multiple potential goals. Although this behavior has been reported before [6, 12, 36], the pattern of compensation is better described as buying more time for decisions. When people are uncertain about the current best option, they delay the decision both by moving towards an intermediate location between the targets and by having a longer reaction time. In contrast, when they are certain about the best option, they initiate movements quickly and aim directly to the selected option. In line with the model predictions, trial by trial reaction time was correlated with the approach direction regardless of the target probability. Longer reaction times were often associated with weak information about the current best option (i.e., strong competition between the desirabilities of the actions). This might suggest that the brain learns to use decision time as a proxy for estimating choice uncertainty (see also [2, 23, 60]).

### 3.2 From signal-detection theory to evidence accumulation to desirability competition

Over the past several years, two prevailing theories have been extensively used to study the computations of uncertainty in decision making; signal-detection theory (SDT) [61, 62] and evidence accumulation models (EAMs) [22, 63–66]. According to SDT theory, a choice is made (e.g., “direction right” vs. “direction left” in a random dot motion task) by comparing a decision variable (DV) against a criterion. Choice uncertainty is determined by the distance of the DV to the criterion. When the evidence strongly supports one option over the other, the distance is larger and the uncertainty about the selected option (i.e., choice uncertainty) is lower [67, 68]. Despite the important contribution of SDT to understanding how uncertainty emerges in decision making, it can predict neither the time it takes to make a decision nor the effects of decision time in choice uncertainty as reported by a series of experimental studies (including our work) [47, 69]. To overcome this inherent limitation of the SDT theory, EAMs were proposed to model a variety of decision tasks. EAMs conceive decision making as a process of noisy accumulation of evidence in favor of the different available options. A decision is made when evidence in favor of one option becomes sufficiently strong (for a review see [70]). The main advantage of EAM over SDT is that it is capable of explaining the association between choice and RT across many domains including perceptual decisions [65, 67, 71, 72], value-based decisions [55, 73, 74], recognition memory tasks [64, 75] and go/no-go tasks [76, 77]. To account for uncertainty in two-choice decisions, evidence is accumulated by two independent counters [78]. The counter that first reaches the amount of evidence required to make a decision determines choice and decision time (i.e., reaction). The level of choice uncertainty is determined by the balance-of-evidence—i.e., the difference in the accumulated evidence between the two counters (the smaller the difference, the higher the degree of uncertainty about the selected option) [21, 22, 24, 40]. Although EAMs have been successfully used to model a variety of cognitive and perceptual decision tasks, they do not include a mechanism for generating motor behavior (e.g., reaches, saccades), with few exceptions, such as a recent study that augmented the drift diffusion model with an action system [15]. However, this embodied model is limited in that it involves only one accumulator, and uses a simplified action model to generate trajectories with constant velocities. Because of that, it cannot make predictions on how choice uncertainty emerges and how it is associated with behavioral measurements, such as reaction time and approach direction. Additionally, another study combined a single motor plan framework with an evidence accumulator to explain intermediate movements in reaching tasks with switching goals prior to movement initiation [13]. The model suggests that intermediate movements reflect a single, deliberate movement plan chosen to maximize task performance based on constraints and motor cost. Despite the fact that the model can predict many key aspects of spatial averaging behavior in tasks with competing goals, it cannot predict the association between reaction time and approach direction that we reported in our study, since it uses only one evidence accumulator to model the uncertainty about the target location. Overall, while EAMs are sufficient for primarily decisions, in which individuals first make a choice and then generate an action to implement the choice, modifications are required for decisions while acting.

Building on evidence accumulation models, we designed and introduced a neurodynamical framework that includes circuitry for generating reaching movements during the decision-making process. It employs a mechanism to integrate information from disparate sources (i.e., spatial location of the target, target probability, effort-cost) dynamically and while acting. However, it is quite different from the traditional EAMs. The competition is based on the relative desirability of the alternative *actions*, instead of the accumulated sensory evidence in favor of one *option* over the others. Desirability is related to the action and provides a more general measure to evaluate an alternative, since it includes information not only about the option itself, but also the action required to achieve that goal. Analogous to the “balance-of-evidence” (i.e., the absolute difference in the state of the two accumulators) in EAMs [24], the momentary degree of choice uncertainty is determined by the “balance-of-desirability” at a given time and state—i.e., the difference between the relative desirability values of the alternative actions. Therefore, the key difference between our model and the normative EAMs is that the locus of uncertainty is over the “action-space” (i.e., relative desirability of the alternative actions) while EAMs attribute uncertainty to the “evidence-space” in perceptual decisions (i.e., difference of accumulated evidence for the chosen option and alternatives) or to the “target-space” in the value-based decisions (i.e., difference between the expected reward between the alternative targets). Additionally, our theory does not assign populations of neurons to the potential targets. Instead, the alternative actions emerge within a distributed neuronal population by integrating information from multiple sources. Consequently, it can easily handle not only binary decisions, but also decisions between multiple competing goals. Traditional EAMs have also been extended to handle decisions with more than two choices in perceptual and value-based decisions [55, 79, 80], although there is an ongoing debate about the right way to generalize these models in multialternative decision tasks (different extensions of the sequential sampling models can lead to significant different behavioral and neurobiological properties) [55, 81, 82]. Finally, the most important difference between the EAMs and our theory is that we can make predictions not only about the decision process, but also about the spatial and the temporal characteristics of the reaching movements. By integrating the neurodynamical framework with stochastic optimal control theory, we can simulate motor behavior (i.e., reaches) from the movement initiation to the final goal location. Hence, we can make predictions on how uncertainty about the current best action influences not only reaction time (i.e., action planning), but also approach direction and velocity of reaches (i.e., action generation) at any given time and state.

How target uncertainty affects the planning of reaching actions is explained by the action competition mechanism implemented in the reach planning field. Therefore, the reach planning field, the spatial sensory input field and the expected outcome field would be sufficient to explain most of the key findings in the current study. However, the stochastic optimal control component is required to explain the characteristics of action execution, such as the signal-dependent noise on the reaching movements, the curved trajectories, the velocity profile of the reaching movements, the association between the initial approach direction and the velocity of movements (see S1 Text) and others. At a glance, the reach cost field, which encodes the effort cost required to implement the single policies, does not seem to have an important role in the model, since both targets are equidistant from the origin. It requires about the same effort to arrive in both targets before movement initiation. However, once departing from the origin, the effort to move towards the two targets varies and depends not only on the current location of the trajectory, but also on the current direction and the movement velocity. Therefore, the reach cost field is also an important component in action execution for “penalizing” effortful actions.

### 3.3 Multiple motor plans versus single motor plan strategy

The key point of our theory is that the brain plans multiple competing actions before deciding which one to execute. Although a growing body of behavioral [6, 10, 16, 31, 36, 41, 83] and neurophysiological studies [14, 84–89] provide evidence that the brain builds parallel actions that compete for selection, other studies argue against this hypothesis showing that premotor areas encode a single action plan [90] and decision and action are separate processes—i.e., planning and execution of action occur after a decision is made [11, 13, 91–96]. According to this theory, the spatial averaging behavior observed in dual-target trials does not necessarily reflect “motor averaging”. Instead, it could be equivalently interpreted as evidence of a single action towards a weighted averaged target location (i.e., visual averaging encoding) [10, 14, 25] or a single optimal and deliberate movement plan to maximize task performance (i.e., performance optimization encoding) [13]. Both of these single action theories are adequate to explain part of the averaging behavior reported in tasks with multiple potential targets. However, they are not sufficient to explain the relationship between approach direction and RT—i.e., RT increases with reaches aimed to an intermediate location regardless of the target probability. If decision and action were two separate cognitive processes, RT would be a function of the time required to plan the single motor action and the time required to initiate that action. In this case, there is no mechanism to explain the effect of approach direction to RT. This effect can be modeled only within two competing modules that integrate sources of information in favor of the alternative actions (see an analogous case for perceptual decisions in [23]).

Recent studies challenge the motor averaging hypothesis presenting evidence against the parallel preparation of multiple competing actions. One of these studies argued that planning and initiation of an action are mechanistically independent [97]. Using a reach-before-you-know task, Haith and colleagues showed that reaction time does not reflect the time at which the competition between the parallel planned actions is resolved—i.e., there is no causal relationship between planning and initiation of actions. Instead, reaction time is determined by an independent initiation process. It is likely that action initiation occurs at a fixed delay after the action planning. However, this study did not account for multiple potential targets that are simultaneously presented prior to movement initiation. Instead, the individuals had to perform center-out reaches to one of eight peripheral targets arranged in a circle, and occasionally to initiate a movement before knowing the actual position of the target. Therefore they did not need to plan multiple actions that compete for selection. Another recent study showed that dynamics associated with competing reach actions, such as grip force, are not averaged [98]. However, this finding does not provide evidence against the hypothesis that the brain generates in parallel actions that compete for selection, and then averages the directions of the individual actions. In other words, we cannot reject the hypothesis that reach parameters, such as direction of reaching movements, are averaged to generate a single initial movement, whereas other parameters, such as grip forces, are not averaged. In fact, the affordance competition hypothesis, which is the base of action competition, states that the brain plans in parallel *partially* prepared actions (i.e., it does not determine all the reach parameters, but only aspects of the actions, such as movement direction) that compete for selection.

Additionally, other psychophysical studies argued that the spatial averaging behavior reflects an optimization based on task constraints and motor cost [11, 13]. When the benefits of intermediate movements are mitigated by reducing the time available to make online corrective movements or by increasing spatial separation between targets, the spatial averaging behavior is abated and reaches more frequently aim to the target locations (for a review see [30]). However, the action competition model presented in our study can explain the lack of spatial averaging behavior in these two conditions. We have showed, in our original study, that desirability is state- and time- dependent, and so the weighted mixture of individual motor policies automatically produces a range of behavior, from winner-take-all (i.e., direct movements to the target locations) to weighted averaging [17, 18]. We also showed that action competition depends on the geometrical configuration of the targets, such as intermediate movements are reduced with the target separation (see Fig 5 in [18]). When two targets are placed in distance, the reaching policies associated with them differ significantly and therefore the competition is often resolved immediately after movement onset, consistent with a neurophysiological study in non-human primates (NHPs) [8]. On the contrary, when two targets are placed in close proximity, the individual reaching policies are similar to each other resulting frequently in intermediate movements (see Fig 5 in [18]). In a similar manner, it is likely that the competition is often resolved prior or immediately after movement onset, when individuals have limited the time to make online corrective movements. Therefore, although spatial averaging is largely abated, when targets are not in close proximity or when time is not allowed to correct movements, this motor behavior can be also explained within the motor encoding strategy as an early resolve of the action competition (even prior to movement initiation). Hence, we can argue that the spatial characteristics of reaching behavior cannot disambiguate whether reaches reflect a single motor plan strategy or a multiple motor plan strategy. Our study explored both the spatial and temporal characteristics of reach behavior showing that approach direction and reaction time are correlated independently of the target probability. This finding cannot be explained without a competition mechanism providing further evidence in favor of the motor encoding strategy.

However, although we believe that our results are best explained by a desirability-driven action competition process, it may be possible that the association between RT and approach direction reflects some kind of target competition process. For instance, the performance optimization theory proposed by Haith et al. in [13] can be extended to model the target selection process as a competition of two noisy internal accumulators (one for each target) which could act on some combination of sensory signals and target expectancies—internal signals of which target is more likely to appear. The two accumulators compete for selection until one of them wins the competition. Then, a single action is initiated towards the average location of the two targets weighted by the activity of the accumulators. The accumulators must take more time to solve the competition when there is uncertain expectancy of the target, because intermediate trajectories have longer RTs. However, in this model the certainty of expectancies should vary with target probability, unless participants get confused about which block they are in, or experience some attentional lapse on some fraction of trials. Within this fraction of trials, the target expectancies from irrelevant conditions might be accumulated, and this would produce longer RTs when this leakage occurs. In other words, the effect could be generated by some attentional noise in setting target expectancies.

### 3.4 Future directions for elucidating the mechanism of choice uncertainty

One of the key findings in our study is that choice uncertainty is represented in the action-space through a desirability driven-competition between motor plans that are encoded in parallel. This finding is based on a neurodynamical theory that adopts the motor averaging hypothesis and captures the motor behavior in reaching movements with goal location uncertainty. According to this theory, the momentary degree of uncertainty is determined by the *balance-of-desirability*—i.e., the difference between the relative desirability values of the competing actions—and is encoded by the same brain areas the plan actions. Brain monitoring studies can further elucidate the mechanisms of choice uncertainty in motor decisions that evolve while acting, in a similar manner that monkey neurophysiological studies investigated choice uncertainty in perceptual decisions. A seminal study provides evidence that neurons in the lateral intraparietal (LIP) area of NHPs encode both the formation of the decision and the uncertainty about the selected action [47]. Importantly, choice, reaction and uncertainty about the selected option are emerged through a common mechanism of bounded evidence accumulation in the LIP area [2]. Similar to this mechanism, our study suggests that the desirability-drive competition between parallel prepare actions unifies choice, reaction time and uncertainty about the selected action in motor decisions that evolve while acting. Future neurophysiological and/or brain-imaging studies will provide better insights on where in the brain and how choice uncertainty is encoded for rapid reaches with multiple competing actions.

### 3.5 Conclusions

In conclusion, when people are uncertain about the best current action, they initiate intermediate movements with longer reaction times. This behavior can be explained within the motor encoding strategy, suggesting that choice uncertainty emerges in the “action-space” through a mechanism of desirability driven-competition between parallel prepared actions, and affects both action planning and action generation.

## 4 Materials and methods

### 4.1 Ethics statement

The University of Southern California review board approved the study protocol and a written informed consent was obtained based on the Declaration of Helsinki.

### 4.2 Experimental setup

Seven right-handed (20–30 years old, 4 men and 3 women) individuals with normal or corrected-to-normal vision participated in this experiment study. A rough sketch of the experimental setup used in this study is shown Fig 2. Participants were seated facing a Phantom Premium 1.5 Haptic Robot (Sensable Technologies, MA) and a computer display, aligned so that the midline of their body was in line with the center of screen and robot. The workspace of the phantom haptic robot forms a hemisphere approximately 30 cm in radius. The participants selected a comfortable position and inserted the right index finger into the endpoint of the tip of the robotic manipulandum. The distance *d*_*subject*_ from the head of the participants to the finger starting position measured along the *y* axis was about 0.30 m. This distance was slightly varied between participants, since we did not use a chin rest or any other restraining device. Hence, there was some movement of the head relative to the screen, but was minimal since the participants were instructed to remain stationary throughout the experiment. The distance from the finger starting position to the screen display *d*_*display*_ was about 0.35 m and was calibrated at the beginning of each session.

The participants were trained to perform rapid reaching movements using the robotic manipulandum. The reaching movements were performed in the horizontal plane (i.e., by restricting the motion of the manipulandum) and translated into movements of a small cursor circle (1.5 cm diameter) in the vertical plane of the computer screen—i.e., reaches towards the screen moved the cursor to the top of the screen, while left and right mapping was preserved. This experimental set up allowed for high temporal and spatial resolution of the hand and finger position as well as a mean to create haptic feedback or altered movement dynamics for future experiments. Control of the phantom robot and the experiment were implemented using the OpenHaptics drivers provided by Sensable technologies, and the Simulation Laboratory (SL) and Real-Time Control Software Package [99] as well as other custom psychophysics software. Control and recording of the phantom state were performed at 500 Hz.

### 4.3 Experimental paradigm

At the start of each trial participants were required to move the cursor to the starting position, located at the origin of our coordinate system, Fig 3A. A fixation cross was then presented at the center of the screen and the participants were instructed to fixate for a short period of time ($\bar{t}=1500\;\text{ms}$, *σ*_*t*_ = 300 ms). During the final 300 ms of fixation, either a single cue was presented on the upper-left or upper-right of the screen or two cues were presented simultaneously in both sides of space. Cues were presented as unfilled circles with 3 cm in radius on a white background. After the fixation offset (go-signal) the participants had to initiate a rapid reaching movement. Once the cursor exceeded a certain trigger threshold (i.e., a virtual wall in the *x* − *z* plane; red discontinuous line in Fig 3A), the single cue or one of the two cues was filled-in black indicating the actual location of the goal. If the participants brought the cursor to the cued target within 1.0 s the trial was considered successful. Trials in which the participants responded before the go-signal or arrived to the cued target after the allowed movement time were aborted and were not used for further analysis. The distance between the origin and the midpoint of the two targets was *d*_*reach*_ = 0.20 m. The target separation distance—i.e., distance between the two target locations—was *d*_*separation*_ = 0.30 m. The trigger threshold distance—i.e., distance of the virtual wall from the origin—was *d*_*threshold*_ = 0.05 m, Fig 3C.

Individuals were familiarized with the task by running a set of training trials that included reaches to single and two targets. Once they felt ready and comfortable with the experimental setup, the actual experiment started. Each participant performed 3 reaching sessions (one training and two tests). The training session involved 40 trials, which were excluded from the analysis, followed by two test sessions; an equiprobable with 80 trials and an unequiprobable with 160 trials. The equiprobable session involved reaches to one (40% of the trials) and two (60% of the trials) targets. In the single-target trials, the cue was shaded blue and was presented equiprobably to the left or right visual field (top row in Fig 3A). In the two-target trials, the cues were also shaded blue and had equal probability of filling-in after the movement onset (bottom row in Fig 3A). The unequiprobable session was similar to the first one with the only difference that one of the cues was always assigned with higher probability in the two-target trials. The “likely” cue was shaded green and had 80% probability of being the correct target, while the alternative cue was shaded red and had 20% probability. The set of target configurations is illustrated in Fig 3B. Individuals were not informed what the coloration indicates and learned the association during the experiment.

### 4.4 Behavioral data analysis

Cubic interpolating splines were used to smooth the reach trajectories and compute the velocity of the movements. The initial approach direction was measured from the direction of the main axis of the covariance ellipse that describes the spatial variation of the cursor from the movement initiation to the goal onset. Reaction time was defined as the time at which the reach velocity exceeded 5% of the maximum velocity. Also, data were pooled across all participants to perform the analyses, unless otherwise specified.

### 4.5 Neurodynamical framework

In the current section, we briefly describe the architecture of the computational framework used to model the reaching experiment. Readers can refer to [17, 18] for more details. The framework combines dynamic neural field (DNF) theory with stochastic optimal control (SOC) theory and includes circuitry for perception, expected outcome, selection bias, effort cost and decision making. Each DNF simulates the dynamic evolution of firing rate activity of a network of 181 neurons over a continuous space with local excitation and surround inhibition. The functional properties of each DNF are determined by the lateral inhibitions within the field and the connections with other fields in the architecture. The projections between the fields are topologically organized—i.e., each neuron *i* in a field drives the activation at the corresponding neuron *i* in the other field. The activity of a DNF evolves over time under the influence of external inputs, local excitation and lateral inhibition interactions as described by Eq (8)

$$\begin{array}{c} \tau \dot{u}(\chi ,t)=-u(\chi ,t)+h+S(\chi ,t)+\int w(\chi -\chi^{\prime })f[u(\chi^{\prime },t)]d\chi^{\prime } \end{array}$$ (8)

where *u*(*χ*, *t*) is the local activity of the DNF at the position *χ* and time *t*, and $\dot{u}(\chi ,t)$ is the rate of change of the activity over time scaled by a time constant *τ*. If there is no external input *S*(*χ*, *t*), the field converges over time to the resting state *H* from the current level of activation. The interactions between the simulated neurons in the DNF are given via the kernel function *w*(*χ* − *χ*′), which consists of both local excitatory and inhibitory components, Eq (9).

$$\begin{array}{c} w\left(\chi -\chi^{\prime }\right)=c_{exc}e^{-\frac{{\left(\chi -\chi^{\prime }\right)}^{2}}{2\sigma_{exc}^{2}}}-c_{inh}e^{-\frac{{\left(\chi -\chi^{\prime }\right)}^{2}}{2\sigma_{inh}^{2}}} \end{array}$$ (9)

where *c*_*exc*_, *c*_*inh*_, *σ*_*exc*_, *σ*_*inh*_ describe the amplitude and the width of the excitatory and the inhibitory components, respectively.

We convolved the kernel function with a sigmoidal transformation of the field so that neurons with activity above a threshold participate in the intrafield interactions, Eq (10).

$$\begin{array}{c} f\left(u\left(\chi \right)\right)=\frac{1}{1+e^{-\beta (u(\chi -))}} \end{array}$$ (10)

The architectural organization of the framework is shown in Fig 6. The “spatial sensory input” field encodes the angular representation of the competing goals in an egocentric reference framework. The expected outcome for reaching to a particular direction centered on the hand position is encoded by the “expected outcome” field (see [17] for more details). In trials with equiprobable targets, the neuronal activity of the populations selective for these targets is about the same (blue Guassian distributions). However, in trials in which one of the targets is more likely than the alternative, the activity of the neuronal population selective for the “green” cue is higher than the activity of the populations which is tuned to the “red” cue. The outputs of these two fields send excitatory projections (green arrows) to the “reach planning” field in a topological manner. The “reach cost” field encodes the effort cost required to implement a sequence of actions towards a particular direction at any time and state. The output of this field sends inhibitory projections (red arrow) to the reach planning field to penalize high-effort actions. The activity of the reach planning field at a given state *x*_*t*_ is sum of the outputs of the fields encoding the location of the target *ν*_*loc*_, the expected outcome *ν*_*outcome*_ and the estimated reach cost *ν*_*cost*_, corrupted by additive noise *ξ* which follows a Normal distribution.

$$\begin{array}{c} S_{action}\left(x_{t}\right)=\eta_{loc}\nu_{loc}\left(x_{t}\right)+\eta_{outcome}\nu_{outcome}\left(x_{t}\right)-\eta_{cost}\nu_{cost}\left(x_{t}\right)+\xi \end{array}$$ (11)

where *η*_*loc*_, *η*_*outcome*_ and *η*_*cost*_ are scalar values that weigh the influence of the spatial sensory input field, the expected outcome field and the reach cost field, respectively, to the activity of action planning field. The values of the model parameters are given in S1 Table. While some studies attempt to find values for these parameters that capture the trade-off that participants make between cost and reward [100, 101], we set the parameter values empirically in order to allow the model to successfully perform the reaching task. The normalized activity of the action planning field describes the “relative desirability” of each policy *π*_*i*_ (*i* = 0, ⋯180)—i.e., it reflects how “desirable” it is to move towards a particular direction *ϕ*_*i*_ with respect to the alternative options.

Each neuron in the reach planning field is linked with a stochastic optimal controller. Once the activity of a neuron *i* exceeds a threshold *γ*, the controller *i* is triggered and generates an optimal policy *π*_*i*_—i.e., sequence of actions towards the preferred direction of the neuron *i*—which is given by minimizing the following cost function:

$$\begin{array}{c} J_{i}(x_{t},\pi_{i})={\left(x_{T_{i}}-Sq_{i}\right)}^{T}Q_{T_{i}}(x_{T_{i}}-Sq_{i})+\sum_{t=1}^{T_{i}-1}\pi_{i}{\left(x_{t}\right)}^{T}R\pi_{i}(x_{t}) \end{array}$$ (12)

where the policy *π*_*i*_ (**x**_*t*_) is a sequence of actions from *t* = 1 to *t* = *T*_*i*_ to move towards the direction *ϕ*_*i*_; *T*_*i*_ is the time required to arrive at the position *q*_*i*_; *q*_*i*_ is the goal-position at the end of the movement and is given as *q*_*i*_ = [*r* cos(*ϕ*_*i*_), *r* sin(*ϕ*_*i*_)], where *r* is the distance between the current location of the hand and the location of the cue which is tuned by the neuron *i*. Additionally, $x_{T_{i}}$ is the state vector at the end of the movement, whereas the matrix *S* picks out the actual position of the hand and the goal-position *q*_*i*_ at the end of the movement from the state vector. Finally, $Q_{T_{i}}$ and *R* define the precision- and the control- dependent cost, respectively. For more details about the optimal control model used in the framework see [17, 18].

The first term of Eq (12) describes the current goal of the controller—i.e., move the hand at a distance *r* from the current location, towards the preferred direction *ϕ*_*i*_ of the neuron *i*. The second term describes the cost (i.e., effort) required for executing the policy *π*_*i*_ (*x*_*t*_). Let’s now assume that *M* neurons are active at a given time *t* (i.e., the activity of *M* neurons is above the threshold *γ*). The framework computes and executes a weighted average of the *M* individual policies *π*_*i*_ to move the hand from the current state *x*_*t*_ to a new one, Eq (13).

$$\begin{array}{c} \pi_{min}(x_{t})=\sum_{i}^{M+i}\nu_{i}(x_{t})\pi_{i}\left(x_{t}\right) \end{array}$$ (13)

where *ν*_*i*_(*x*_*t*_) is the normalized activity of the neuron *i* (i.e., the relative desirability value) at the state *x*_*t*_. Because the desirability is time- and state- dependent, the weighted mixture of the individual policies produces a range of behavior, from winner-take-all (i.e, direct reaching to a target) to spatial averaging.

To handle contingencies, such as perturbations and incoming new information (e.g., changes on the number of targets, target probabilities, expected rewards, etc) and effects of noise, the framework implements a widely used technique in stochastic optimal control known as “receding horizon” [27, 28]. It has been utilized in motor neuroscience studies for modeling human postures and joint movements [102], learning mechanisms in path tracking tasks [103], trajectory planning in voluntary human arm movements [104], grasping objects with position uncertainty [105] and others. According to the receding horizon control, the framework executes only the initial portion from the sequence of actions for a short period of time *k* (*k* = 10 in our study) and then recomputes the individual optimal policies *π*_*i*_(*x*_*t*+*k*_) from time *t* + *k* to *t* + *k* + *T*_*i*_ and remixes them. This approach continues until the hand arrives to one of the targets.

## Supporting information

S1 Fig Averaging individual motor policies. **A**: Instantaneous velocities generated by the two individual policies *π*_*i*_(**x**_*t*_) (gray trace) and *π*_*j*_(**x**_*t*_) (black trace) for reaching to the left and the right target, respectively. Policies are computed for *t* = 100 time-steps, but velocity vectors are illustrated every 10 time-steps for visualization purposes. By averaging the individual policies in an equiprobable trial, using their desirability values as weighted factor, we get a spatial averaging policy *π*_*avg*_(**x**_*t*_) (blue trace) that generates a reaching movement towards an intermediate location between the targets. Note that for the simulated experiments presented in the main manuscript, the derived averaging policy uses only the first *k* = 10 time-steps of the individual policies. Then, new control policies are recomputed from the current state until the trajectory arrives to one of the targets (receding horizon strategy). **B**: Velocity profile of the trajectories generated by the individual policies (black and gray traces) and the weighted average policy (blue trace) for 100 time-steps. Note that movement velocity decreases when averaging the two individual policies.
(TIF)

S2 Fig Reaching velocity profile in simulated tasks. **A**: Maximum velocity before the target onset for different target probabilities in simulated experiments. **B**: Maximum velocity as a function of the initial approach direction from the simulated equiprobable (blue) and unequiprobale (green) trials. The motor-averaging hypothesis predicts that target uncertainty influences the movement velocity by slowing down the reaches. It also predicts a direct association between the initial approach direction and movement velocity, such as reaches that are aimed towards an intermediate location are slower than reaches that are launched directly to one of the targets, regardless of the target probabilities. Error bars correspond to standard error (SE) and solid lines show the polynomial regression fitting (quadratic in panel A and 4^*th*^ order polynomial in panel B).
(TIF)

S3 Fig Reaching velocity profile in human tasks. Similar to S2 Fig, but for the human reaching movements **A**: Maximum velocity before the target onset for different target probabilities. **B**:Maximum velocity as a function of the initial approach direction in the equiprobable (blue) and unequiprobale (green) trials. Error bars correspond to standard error (SE) and solid lines show the polynomial regression fitting (quadratic in panel A and 4^*th*^ order polynomial in panel B). The human findings are in favor of the motor-averaging hypothesis.
(TIF)

S4 Fig Reaction time for different single target locations. Reaction time as a function of the distance of each target from the midline computed in single-target trials across 3 participants. Error bars correspond to standard error (SE). We found no significant association between reaction time and target location (p-value > 0.197 of the regression coefficients for linear and curvilinear regression analysis).
(TIF)

S1 Text Action competition influences movement velocity. The motor averaging hypothesis predicts the velocity profile of the reaching movements.
(PDF)

S1 Table Model parameters. The parameters of the neurodynamical framework.
(PDF)
