# Supplementary figures and images for: Action planning and control under uncertainty emerge through a desirability-driven competition between parallel encoding motor plans
Source: PLoS Comput Biol. 2021 Oct 1;17(10):e1009429. doi: 10.1371/journal.pcbi.1009429 (PMC8513832; doi:10.1371/journal.pcbi.1009429)

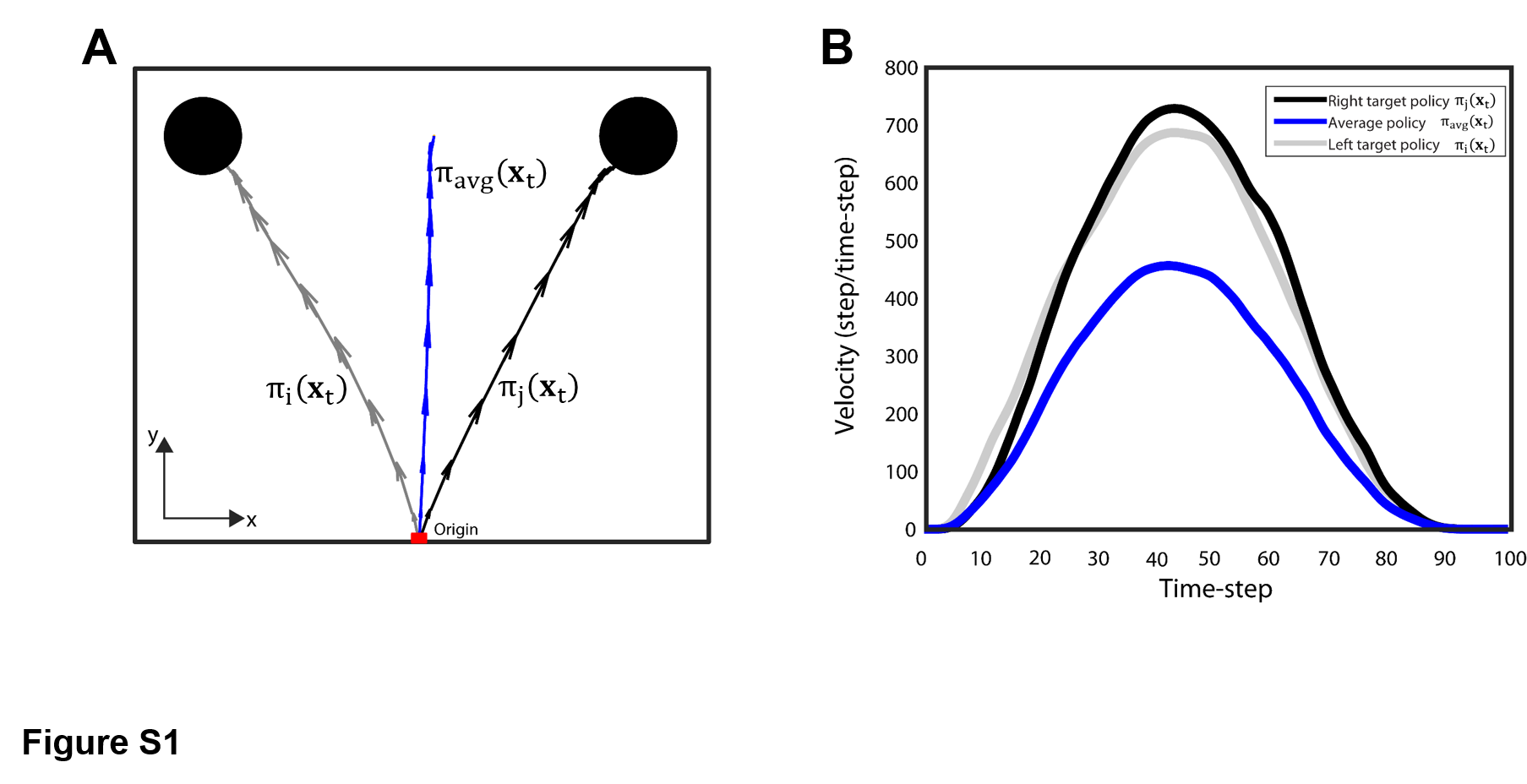

Supplement: S1 Fig — A: Instantaneous velocities generated by the two individual policies πi(xt) (gray trace) and πj(xt) (black trace) for reaching to the left and the right target, respectively. Policies are computed for t = 100 time-steps, but velocity vectors are illustrated every 10 time-steps for visualization purposes. By averaging the individual policies in an equiprobable trial, using their desirability values as weighted factor, we get a spatial averaging policy πavg(xt) (blue trace) that generates a reaching movement towards an intermediate location between the targets. Note that for the simulated experiments presented in the main manuscript, the derived averaging policy uses only the first k = 10 time-steps of the individual policies. Then, new control policies are recomputed from the current state until the trajectory arrives to one of the targets (receding horizon strategy). B: Velocity profile of the trajectories generated by the individual policies (black and gray traces) and the weighted average policy (blue trace) for 100 time-steps. Note that movement velocity decreases when averaging the two individual policies. (TIF) [file pcbi.1009429.s001.tif]

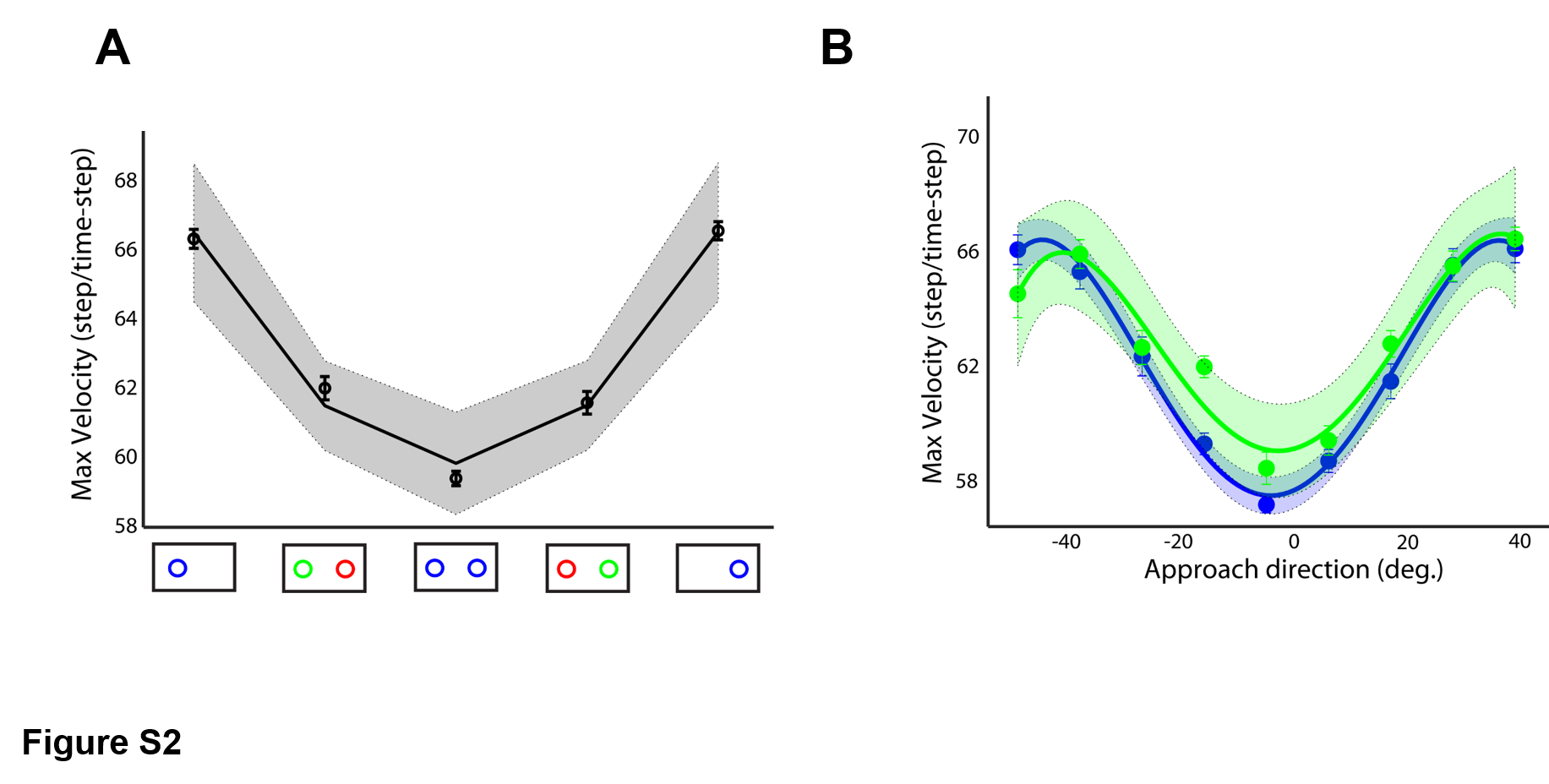

Supplement: S2 Fig — A: Maximum velocity before the target onset for different target probabilities in simulated experiments. B: Maximum velocity as a function of the initial approach direction from the simulated equiprobable (blue) and unequiprobale (green) trials. The motor-averaging hypothesis predicts that target uncertainty influences the movement velocity by slowing down the reaches. It also predicts a direct association between the initial approach direction and movement velocity, such as reaches that are aimed towards an intermediate location are slower than reaches that are launched directly to one of the targets, regardless of the target probabilities. Error bars correspond to standard error (SE) and solid lines show the polynomial regression fitting (quadratic in panel A and 4th order polynomial in panel B). (TIF) [file pcbi.1009429.s002.tif]

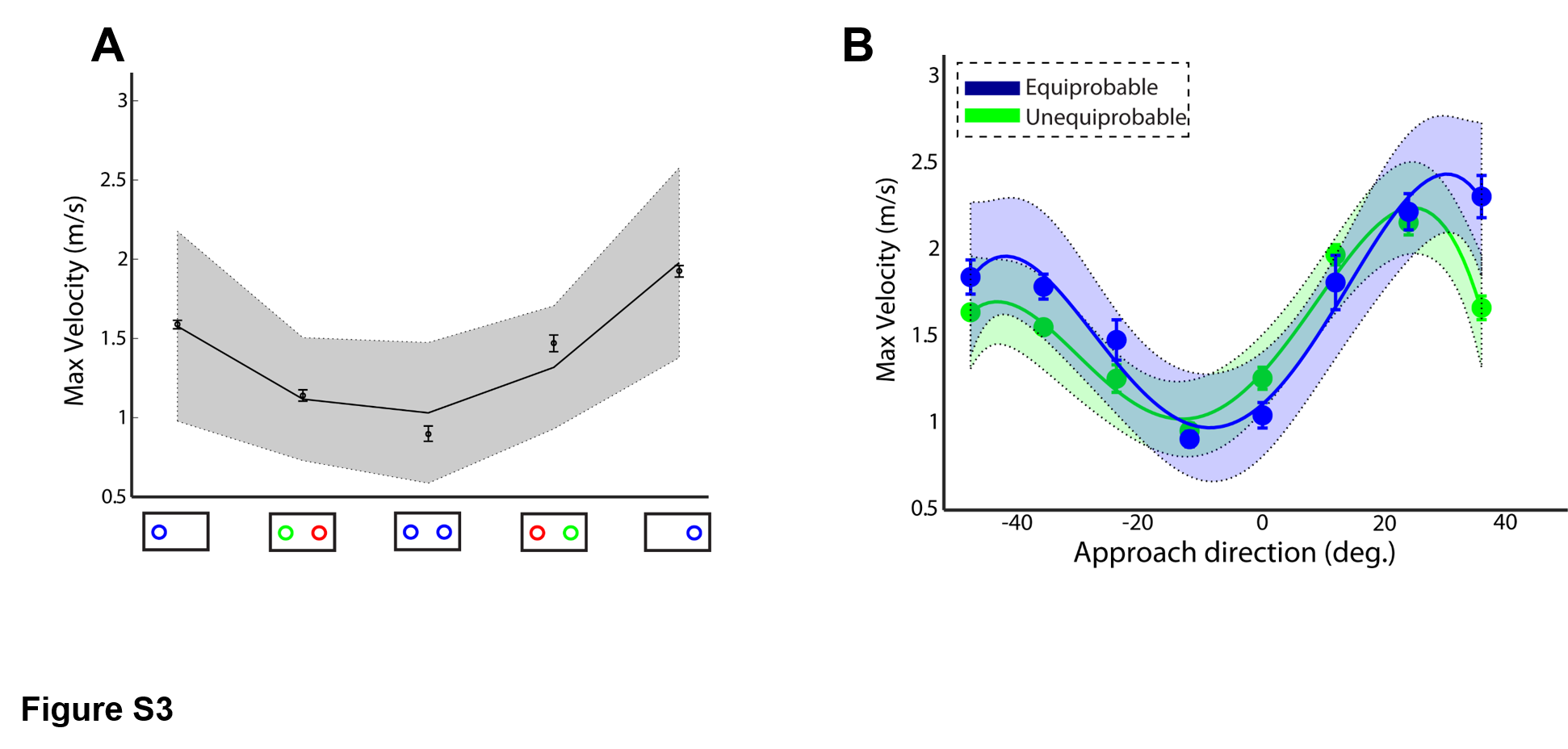

Supplement: S3 Fig — Similar to S2 Fig, but for the human reaching movements A: Maximum velocity before the target onset for different target probabilities. B:Maximum velocity as a function of the initial approach direction in the equiprobable (blue) and unequiprobale (green) trials. Error bars correspond to standard error (SE) and solid lines show the polynomial regression fitting (quadratic in panel A and 4th order polynomial in panel B). The human findings are in favor of the motor-averaging hypothesis. (TIF) [file pcbi.1009429.s003.tif]

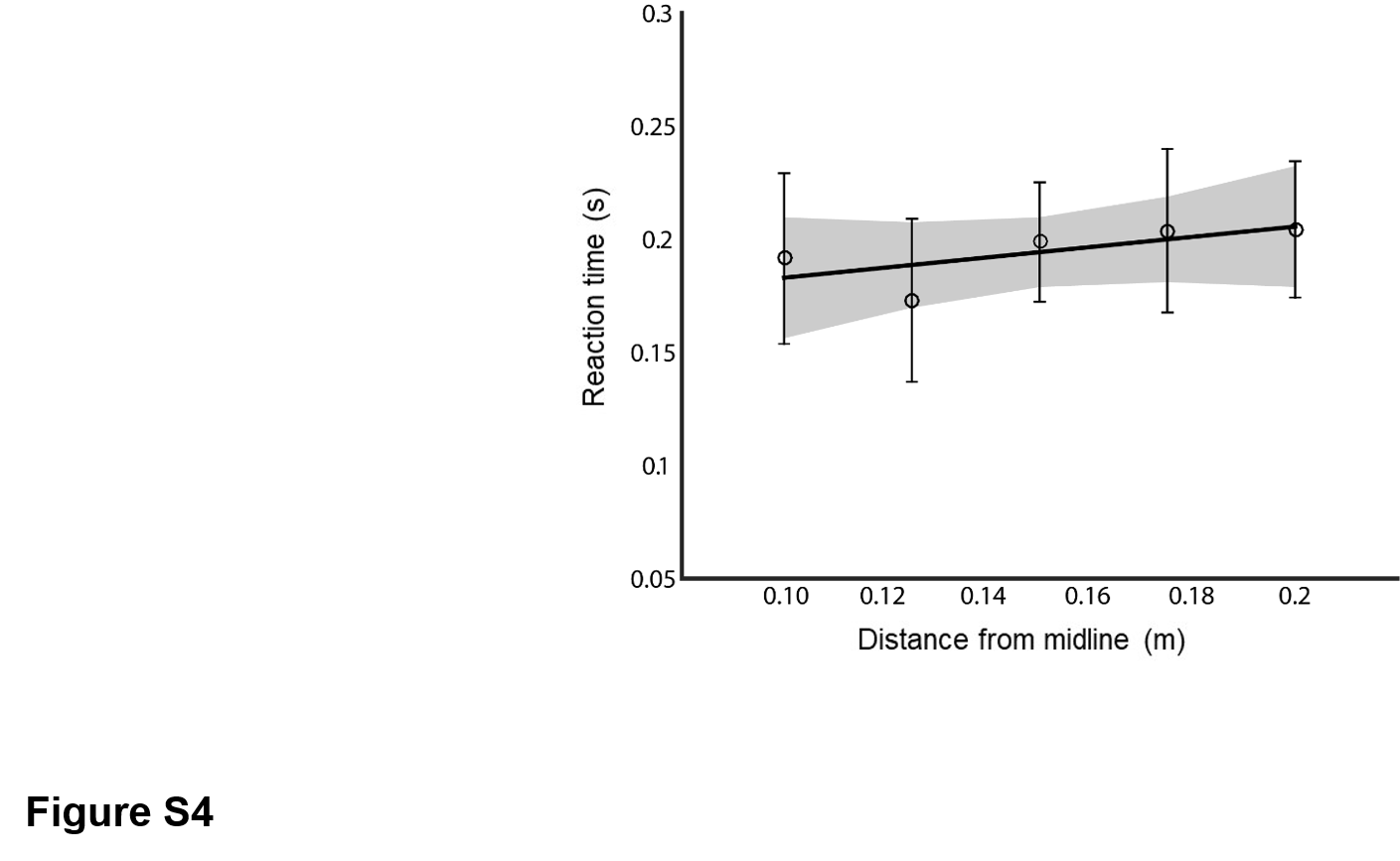

Supplement: S4 Fig — Reaction time as a function of the distance of each target from the midline computed in single-target trials across 3 participants. Error bars correspond to standard error (SE). We found no significant association between reaction time and target location (p-value > 0.197 of the regression coefficients for linear and curvilinear regression analysis). (TIF) [file pcbi.1009429.s004.tif]
