# Supplementary material for: Action planning and control under uncertainty emerge through a desirability-driven competition between parallel encoding motor plans
Source: PLoS Comput Biol. 2021 Oct 1;17(10):e1009429. doi: 10.1371/journal.pcbi.1009429 (PMC8513832; doi:10.1371/journal.pcbi.1009429)
Supplement: S1 Text — The motor averaging hypothesis predicts the velocity profile of the reaching movements. (PDF) [file pcbi.1009429.s005.pdf]

S1 Text

In the current study we showed that action planning and execution under uncertainty emerge through a desirability driven competition between parallel encoding motor plans. This is consistent with the affordance competition hypothesis, according to which reaching movements are generated as a weighted average of individual motor actions [1–3]. One of the novel findings in our study is that reaction time is correlated with the approach direction, such as reaching movements that are launched towards an intermediate location exhibit longer reaction time than reaches that aim directly to the targets. Importantly, this association does not depend on the target probability.

To further explore how this strategy affects reach behavior, let's consider a dual-target trial from the equiprobable session with two potential targets presented simultaneously on both hemifields. For simplicity reason, we assume that only two reach planning neurons i and j associated with the left and right target, respectively, are active prior to movement initiation. These active neurons trigger the corresponding optimal controllers that generate two policies (i.e., sequence of actions) $\pi_i(\mathbf{x}_t) = [u_i(t_1), u_i(t_2), ...u_i(t_{end})]$ and $\pi_j(\mathbf{x}_t) = [u_j(t_1), u_j(t_2), ...u_j(t_{end})]$ towards the preferred direction of the neurons i and j (see Materials and Methods section for more details). These two control policies produce direct reaching movements towards the locations of the two potential targets. S1A Fig illustrates the instantaneous velocities of the two reaching policies (black and gray traces) every 10 time-steps (for better visualization) from the origin (red box) to the target locations (black circles). The velocity profiles of the two reaching trajectories, which would be generated if the model implemented the corresponding reaching policies π_i and π_j are shown in S1B Fig (black and gray traces). These two policies cannot be executed simultaneously. Instead, the model computes and executes an average of these two policies weighted by their relative desirability values (i.e., ν_i, ν_j),

$\pi_{avg}(\mathbf{x}_t) = \nu_i(\mathbf{x}_t)\pi_i(\mathbf{x}_t) + \nu_j(\mathbf{x}_t)\pi_j(\mathbf{x}_t)$ (avg = average). The model uses a receding horizon strategy to recalculate the average policy π_{avg} every 10 steps until the trajectory arrives to one of the two target locations (see materials and methods section for more details). Because policies have about the same desirability values before the movement initiation (equiprobable trial), the average policy $\pi_{avg}(\mathbf{x}_t)$ produces a reaching movement towards an intermediate location between the two potential targets (blue trace in S1A Fig). Notably, the velocity of the executed reaching movement (blue trace) is lower than the velocity of the movements that the individual policies would be generated if they were implemented, S1B Fig. This finding indicates that averaging individual policies produces slower movements than planning and executing a single policy towards one of the potential target locations (single-target trials). This predicts that target location uncertainty influences also the velocity of the reaching movement.

To test this hypothesis, we computed the velocities of *simulated* trajectories from reach initiation to target onset (i.e., when the actual goal was revealed) for the equiprobable and unequiprobale sessions. S2A Fig illustrates the peak of the simulated velocity of the movements prior to target onset for different target probabilities. Consistent with the hypothesis, the model predicts the association between reach velocity and target uncertainty - i.e., the higher the uncertainty about the goal location the slower the velocity of the reaching movement (best fit quadratic regression model: R-square = 0.988, p-value = 0.0123 of the quadratic coefficient). A trial-by-trial analysis revealed also an association between approach direction and movement velocity S2B Fig. In particular, reaches that were launched to an intermediate location between the potential targets were slower than reaches that were aimed directly to one of the two targets regardless of the target probability (best fit 4th order polynomial regression fitting model for both sessions: R-square $>$ 0.948, p-value $<$ 0.00795). This prediction is similar to the association between the initial

approach direction and reaction time that we showed in the main document. Overall, the model predicts that both planning (i.e., reaction time) and execution (i.e., movement velocity) are influenced by the target uncertainty in a predictable manner - reaching movements that launch towards an intermediate location are slower and have longer reaction times than reaching movements that aim directly to one of the two potential target locations.

Consistent with the model prediction, we found that participants moved slower when there was more uncertainty about the actual target location, S3A Fig (quadratic regression model: R-square = 0.931, p-value = 0.06 of the quadratic coefficient). Importantly, a trial-by-trial analysis showed that movement velocity and approach direction were correlated in both sessions. S3B Fig illustrates the peak of the movement velocity as a function of the approach direction across all participants and trials separately for the equiprobable and the unequiprobable sessions. Reaches that were launched towards an intermediate location between the potential targets were slower than reaches that were aimed directly to one of the two targets regardless of the target probability (best fit 4th order polynomial regression fitting model for both sessions: R-square > 0.966, p-value < 0.015). These findings are in favor of the motor-averaging hypothesis suggesting also that the movement velocity can be used an easy-to-measure proxy of choice uncertainty, such as the higher the uncertainty about the current best action the slower the reaching movement.

References

1. Cisek P. Cortical mechanisms of action selection: the affordance competition hypothesis. *Philos Trans R Soc Lond B Biol Sci.*, 362(1485):1585–1599, 2007.

2. Cisek P. Making decisions through a distributed consensus. *Curr Opin Neurobiol.*, 22(6):927–936, 2012.

3. Gallivan JP, Barton KS, Chapman CS, Wolpert DM, and Flanagan JR. Action plan co-optimization reveals the parallel encoding of competing reach movements. *Nat Commun.*, 6(7428), 2015.
