# Supplementary material for: Action planning and control under uncertainty emerge through a desirability-driven competition between parallel encoding motor plans
Source: PLoS Comput Biol. 2021 Oct 1;17(10):e1009429. doi: 10.1371/journal.pcbi.1009429 (PMC8513832; doi:10.1371/journal.pcbi.1009429)
Supplement: S1 Table — The parameters of the neurodynamical framework. (PDF) [file pcbi.1009429.s006.pdf]

S1 Table

Model parameters		
**Parameters**	**Description**	**Value**
n_{loc}	visual input gain	6.2
n_{cost}	action cost input gain	-0.1
$n_{outcome}$	expected outcome input gain	0.55
γ	action threshold	0.65

Spatial sensory input field & Expected outcome field parameters		
**Parameters**	**Description**	**Value**
τ	time constant	5.0
c_{exc}	amplitude of excitatory portion of weight kernel	0
c_{inh}	amplitude of inhibitory portion of weight kernel	0
σ_{exc}	width of excitatory portion of weight kernel	5.0
σ_{inh}	width of inhibitory portion of weight kernel	40.0
h	resting activity level	-5.0
q	noise level	0.25
σ_q	width of noise kernel	5.0
β	steepness of sigmoid activity function	1.0

Reach planning field		
**Parameters**	**Description**	**Value**
τ	time constant	5.0
c_{exc}	amplitude of excitatory portion of weight kernel	5
c_{inh}	amplitude of inhibitory portion of weight kernel	12.0
σ_{exc}	width of excitatory portion of weight kernel	5.0
σ_{inh}	width of inhibitory portion of weight kernel	40.0
h	resting activity level	-5.0
q	noise level	0.25
σ_q	width of noise kernel	5.0
β	steepness of sigmoid activity function	1.0
